# Future productivity and phenology changes in European grasslands for different warming levels: implications for grassland management and carbon balance

**DOI:** 10.1186/s13021-017-0079-8

**Published:** 2017-05-04

**Authors:** Jinfeng Chang, Philippe Ciais, Nicolas Viovy, Jean-François Soussana, Katja Klumpp, Benjamin Sultan

**Affiliations:** 1 Laboratoire des Sciences du Climat et de l’Environnement, UMR8212, CEA-CNRS-UVSQ, 91191 Gif-Sur-Yvette, France; 2 Sorbonne Universités (UPMC), CNRS-IRD-MNHN, LOCEAN/IPSL, 4 Place Jussieu, 75005 Paris, France; 3 INRA, UAR0233 CODIR Collège de Direction. Centre-Siège de l’INRA, Paris, France; 4 Grassland Ecosystem Research Unit, French National Institute for Agricultural Research (INRA), 63100, Clermont-Ferrand, France

**Keywords:** European grassland, Grassland management, Phenology, Warming levels, Climate change

## Abstract

**Background:**

Europe has warmed more than the global average (land and ocean) since pre-industrial times, and is also projected to continue to warm faster than the global average in the twenty-first century. According to the climate models ensemble projections for various climate scenarios, annual mean temperature of Europe for 2071–2100 is predicted to be 1–5.5 °C higher than that for 1971–2000. Climate change and elevated CO_2_ concentration are anticipated to affect grassland management and livestock production in Europe. However, there has been little work done to quantify the European-wide response of grassland to future climate change. Here we applied ORCHIDEE-GM v2.2, a grid-based model for managed grassland, over European grassland to estimate the impacts of future global change.

**Results:**

Increases in grassland productivity are simulated in response to future global change, which are mainly attributed to the simulated fertilization effect of rising CO_2_. The results show significant phenology shifts, in particular an earlier winter-spring onset of grass growth over Europe. A longer growing season is projected over southern and southeastern Europe. In other regions, summer drought causes an earlier end to the growing season, overall reducing growing season length. Future global change allows an increase of management intensity with higher than current potential annual grass forage yield, grazing capacity and livestock density, and a shift in seasonal grazing capacity. We found a continual grassland soil carbon sink in Mediterranean, Alpine, North eastern, South eastern and Eastern regions under specific warming level (SWL) of 1.5 and 2 °C relative to pre-industrial climate. However, this carbon sink is found to saturate, and gradually turn to a carbon source at warming level reaching 3.5 °C.

**Conclusions:**

This study provides a European-wide assessment of the future changes in productivity and phenology of grassland, and their consequences for the management intensity and the carbon balance. The simulated productivity increase in response to future global change enables an intensification of grassland management over Europe. However, the simulated increase in the interannual variability of grassland productivity over some regions may reduce the farmers’ ability to take advantage of the increased long-term mean productivity in the face of more frequent, and more severe drops of productivity in the future.

**Electronic supplementary material:**

The online version of this article (doi:10.1186/s13021-017-0079-8) contains supplementary material, which is available to authorized users.

## Background

Global mean surface temperature (GMST) has increased since the late nineteenth century, and each of the past three decades has been warmer than all the previous decades [1]. If greenhouse gas (GHG) emissions continue unabated, GMST will continue to rise over the twenty first century [2]. Continual warming is predicted by the World Climate Research Program ‘Climate Model Inter-comparison Project’ CMIP3 [3] and the more recent CMIP5 climate projections [4]; see [5] for the comparison between CMIP3 and CMIP5. For Europe, annual mean temperature for 2071–2100 is predicted to be 1–5.5 °C higher than that for 1971–2000. Specifically, a regional warming of 3–4.5 °C in the EU-FP6 ENSEMBLES multi-model ensemble for the scenario A1B of the Special Report on Emission Scenario (SRES) [6, 7], and of 1–4.5 and 2.5–5.5 °C in the new regional climate models ensemble EURO-CORDEX for the Representative Concentration Pathways RCP4.5 and RCP8.5 respectively [8]. Compared to pre-industrial climate, Europe has warmed more than the global average (land and ocean), and slightly more than global land temperature [9]. The decade from 2006 to 2015 was 1.5 °C warmer in Europe, against 0.83–0.89 °C at global scale. In the future, Europe is also projected to continue to warm faster than the global average (land and ocean; [9]). Spatially, greater warming in Southern Europe and towards the northeast is predicted in all climate scenarios. For annual precipitation, an increase in Central Europe and Northern Europe and a decrease in Southern Europe are predicted by regional climate change projections [8], i.e. a dryer Southern Europe and a wetter Northern Europe. In addition, more heavy precipitation events, extended dry spells and more heat waves are predicted in some regions of Europe in the future [8].

Grassland ecosystems cover 56.8 million ha (13.2%) of the land area in the EU-27 [10]. Most of these grasslands are used to feed animals, either directly by grazing or indirectly by grass harvest (mowing). Accounting for multiple drivers of climate change, rising CO_2_, nitrogen addition, and land cover and management intensity changes, a recent study suggested that European grasslands acted as a C sink in the past five decades (15 ± 7 g C m^−2^ year^−1^ for 1961–2010; Chang et al. [11, 12]). In the future, emission scenarios and predicted climate change can profoundly impact the production (annual amount and seasonality), management (grazing timing and animal carrying capacity), and the carbon balance and non-CO_2_ greenhouse gas emissions (i.e., CH_4_ and N_2_O) of grassland ecosystems.

The response of grasslands to climate change is complex as it implies interactions with water availability, nutrients, soil vegetation and management intensity [13]. Elevated CO_2_ concentration has the dual effect of increasing leaf photosynthesis and leaf area index, and reducing stomatal conductance. These effects at ecosystem-scale result in an increase in above-ground dry matter (DM) production of grassland [14–16], increase water-use efficiency [17] and reduce the consumption of soil moisture by plant transpiration [18]. However, trends and variability in temperature and precipitation, as well as possible nitrogen limitations, all interact with the effects of elevated CO_2_ to determine actual changes in grassland productivity [18–20]. In addition, there could be some risks for surviving/adapting of the grass species under rapid climate change, given the fact that the projected rate of climate change far outweighs the rate of niche change in grasses [21].

Climate change and elevated CO_2_ concentration are anticipated to affect grassland management and livestock production in Europe [22, 23] with economic consequences that are yet to be sufficiently assessed [24]. The response of grazing systems may also vary markedly across European regions and with pasture type (e.g., [25, 26]). Under projected future conditions, Graux et al. [27] simulated an increased inter-annual and seasonal variation of grassland production at 12 contrasted French grassland sites using the Pasture Simulation model (PaSim). They also predicted to a significant increase in summer drought risk. Using a probabilistic risk analysis, Van Oijen et al. [28] estimated the drought vulnerability and risk of the carbon and water balance across Europe based on vegetation models, including PaSim for grassland ecosystems. Projections of climate change impacts on European grassland productivity are mostly based on local modeling studies that require many local variables, and this limits the up-scaling to regional or continental scale (e.g., [29]). Grid-based process-based vegetation models with equations representing biogeochemical and biophysical mechanisms have the advantage of being applicable from local to continental scale. This type of mechanistic models is increasingly used for global impact studies on agricultural productivity and terrestrial carbon fluxes (e.g. ISIMIP, http://www.isi-mip.org). However, most of the existing grid-based process-based ecosystem models currently simulate managed grassland either as natural grassland or as a sort of cropland with intensive harvest. Therefore, in order to make more realistic predictions of the impact of future climate change on European grassland, management processes must be included in grassland ecosystem models.

In the COP 21 UN Climate change conference, the Paris Agreement [30] recalls the article 2 of the United Nations Framework Convention on Climate Change [31], implementing the aim of *“holding the increase in the global average temperature to well below 2 * *°C above pre*-*industrial levels and to pursue efforts to limit the temperature increase to 1.5 * *°C above pre*-*industrial levels, recognizing that this would significantly reduce the risks and impacts of climate change”*. In this context, the objective of this study is to predict changes in productivity, growing season, management and carbon fluxes of European grassland when global warming reaches these specific warming levels (SWL) of 1.5, 2 °C relative to pre-industrial climate. The impacts of high warming levels of 3 and 3.5 °C are also investigated to quantify the risks that could be avoided by the Paris agreement. Last, a sensitivity test is carried out to investigate the impact of fixed vs. adjustable livestock intensification on grassland carbon fluxes under climate change and the rising CO_2_ concentration.

## Methods

### Model description

ORCHIDEE is a process-based ecosystem model built for simulating carbon cycling in ecosystems, and water and energy fluxes from site-level to global scale [32–34]. ORCHIDEE-GM is a version specifically developed to integrate the management of grassland with two options [35]. Either the model can be forced by observed animal density for grazing or by observed harvest time for forage removals, or it can calculate the *optimal* densities and practices that maximize the use of ecosystem productivity. The equations describing management in ORCHIDEE-GM are derived from PaSim [36]; Vuichard et al. [37–39]. ORCHIDEE-GM version 1 was evaluated and some of its parameters calibrated at 11 European grassland sites representative of a range of management practices, with eddy covariance net ecosystem exchange (NEE) and biomass measurements. The model showed capability to simulate net biome productivity (NBP; i.e. the C balance) of these managed grasslands [35] even though it does not include an explicit nitrogen cycle interacting with the carbon cycle. Chang et al. [40] further added a new parameterization to describe an adaptive management strategy of farmers who react to a climate driven change of previous-years’ productivity. The positive effect of N addition on grass photosynthesis, and thus on subsequent ecosystem carbon balance, are parameterized with a simple empirical function calibrated from literature estimates (version 2.1; [40]. At continental scale, ORCHIDEE-GM v2.1 was applied over Europe to calculate the spatial pattern, recent trends and interannual variability of potential productivity (the productivity corresponding to an optimal management practice that maximizes livestock densities). This version was further used to simulate NBP and NBP trends over European grasslands during the last five decades at a spatial resolution of 25 km [11, 12]. In this study, ORCHIDEE-GM v2.2 is released with (1) an update of the general parameterizations from ORCHIDEE Trunk.rev3623 (https://forge.ipsl.jussieu.fr/orchidee/browser/trunk#ORCHIDEE), and (2) a new parameterization incorporated specific management strategies.

### Specific management strategies in ORCHIDEE-GM v2.2

Grazing when the air temperature is below freezing point 0 °C (frost-grazing) and grazing on grassland covered by snow (snow-grazing) increase farmers labor as additional energy is needed for maintenance and feed supplementation. Furthermore, as for wet soil described below, grazing of snow-covered ecosystems often leads to grassland degradation and trampling due to concentration of animals around feed points. Therefore, frost-grazing and snow-grazing are usually avoided by farmers given the fact that they are not economically efficient.

Moreover, livestock trampling over wet soil (wet-grazing) causes excessive soil compaction, represented by the increase in bulk density and soil strength, and the decrease in water infiltration rate [41]. Excessive soil compaction could result in soil degradation and further impact the sustainability of pasture productivity. Thus in practice, farmers tend to avoid grazing when the soil is too wet.

Here, the following new set of management rules has been incorporated into ORCHIDEE-GM v2.2 to represent the above limitations on grazing:

1. Grazing stops if daily mean air temperature drops below 0 °C.
2. Grazing stops when grassland is covered by snow, and for three consecutive days after snow melt. To simulate snow cover, the mechanistic intermediate-complexity snow scheme (ISBA-ES; Boone and Etchevers [42] has been implemented in ORCHIDEE-GM v2.2 (updated with ORCHIDEE Trunk.rev3623), which improves the representation of snow processes such as snowmelt timing [43].
3. Grazing stops when soil becomes too wet (i.e., soil moisture close to saturation), and can only be resumed after at least 10 consecutive days after the stop to avoid soil degradation due to trampling. Here, wet soil condition is defined in the model by daily mean soil moisture content (*mc*) of topsoil (0–9 cm in this study) being close to saturation:

1 $$mc \ge mc_{sat} - \Delta_{crit}$$

where *mc*_*sat*_ is the saturated soil moisture content derived from Carsel and Parrish [44]; the threshold *mc*_*sat*_ *−* ∆_*crit*_ represents an empirical value determining wet soil conditions; the value of ∆_*crit*_ is set to 0.05 m^3^ m^−3^. To avoid grazing cessation during instantaneous soil saturation caused by discrete heavy precipitation, grazing only stops when wet soil conditions last for over 3 days during a running window of the previous 5 days. The 0–9 cm mean soil moisture is chosen here given the fact that (1) instead of the two-layer simple bucket used in previous ORCHIDEE-GM versions, ORCHIDEE-GM v2.2 here includes the more complex 11-layer soil–water diffusion scheme (implemented in ORCHIDEE Trunk.rev3623; De Rosnay et al. [45, 46], D’Orgeval [47, 48] to be able to simulate soil moisture in different soil layers from 1 mm depth for the first layer down to 2 m depth for the 11th layer (e.g., the top 5, 6, 7 layers of the soil represent 0–5, 0–9, and 0–19 cm soil depth respectively), (2) within the above vertical profile that model used, 9 cm is the most realistic depth impacted by livestock trampling (around 10 cm in practice). A sensitivity test using the top 6 (0–9 cm) or 7 layers (0–19 cm) of soil moisture as topsoil *mc* only shows marginal difference in both the *mc* value and the impact on grazing (data not shown).

### Biological *potential* productivity, grazing capacity, and optimal livestock density

ORCHIDEE-GM simulates the two practices through which managed grassland provides grass biomass for livestock: mowing and grazing. Model rules automatically determine the frequency and magnitude of mowing (harvests) events in each grid cell [39], which simulates a potential (maximum) productivity. Here, we define grassland *potential* productivity as the annual maximum production of forage from mown grassland (*Y*_*pot*_, kg of DM per hectare of mown grasslands). In terms of grazing, due to the impact of livestock on grass growth through trampling, defoliation (i.e., biomass intake) etc., and because grassland cannot be grazed continuously over the whole vegetation period, biomass production has to meet minimal conditions before animals can be applied (e.g., 18 kg DM intake day^−1^ LU^−1^; LU, livestock unit; [12]. Accordingly, thresholds of shoot biomass (Vuichard et al. [38]; updated in [37], snow, temperature and soil moisture conditions (see “Specific management strategies in ORCHIDEE-GM v2.2” for detail) are set for starting, stopping and resuming grazing. Consequently, the model estimates the number of grazing days (*N*_*grazing*_) and barn days (i.e., off-site period; e.g., *N*_*barn*_ = 365 − *N*_*grazing*_ for each year) for each grid and each time period (month or year). The grazing capacity for any time period (*C*_*grazing,i*_; unit: LU days ha^−1^) can be calculated as:

2 $$C_{grazing,i} = S_{opt,i} \times N_{grazing,i}$$

where *S*_*opt,i*_ is the optimal instantaneous animal stocking rate at time period *i* (LU per hectare of grazed grassland; *i* can be 1 day, 1 month, one season or 1 year); *N*_*grazing,i*_ is the number of days when animals are grazing the pasture.

Meanwhile a set of rules allowing the simulation of idealized self-sufficient herbage-based ruminant livestock farm [39] have been introduced into ORCHIDEE-GM v2.1 [40]. These rules are based upon two assumptions, (1) within each grid cell, livestock can only be fed by herbage (i.e. crop-fed animals are not considered), and (2) the use of grassland production is maximized in each grid cell, by calculating the mix of mowing and grazing that maximizes the number of animals in that grid cell. Under these assumptions, the optimal instantaneous animal stocking rate, *S*_*opt*_, the optimal proportion of grazed vs. mown grasslands, *F*_*opt*_ (within [0,1]), and thus an optimal livestock density, *D*_*opt*_ (LU per hectare of total grassland) are calculated for each grid cell using the optimization algorithm of Vuichard et al. [39]. We further incorporated specific rules that were incorporated into ORCHIDEE-GM v2.1, in order to model adaptive management in response to climate variability. In other words, *S*_*opt*_, *F*_*opt*_, and *D*_*opt*_ change in response to climate-driven changes in the grassland productivity of previous years [40].

### Defining active growing season and grazing season

A positive NPP defines grass growth and the accumulation of biomass, which further impacts grazing practice. To detect a temporal shift (i.e., advance or delay of the active growing season and grazing season), we use four indicators in this study. The first one is the length of the active growing season in each grid cell (*L*_*growing*_, unit: days per year) defined as the number of days per year when NPP is higher than 1 g C m^−2^ day^−1^. The second one is the beginning of the active growing season in each grid cell (*B*_*growing*_, unit: day of year) being the first day of the year when (1) the grassland NPP is higher than 1 g C m^−2^ day^−1^, and (2) a positive NPP trend (i.e., growth rate is larger than 0.05 g C m^−2^ day^−2^) is found for the following 10 days. The third one is the length of grazing season in each grid cell (*L*_*grazing*_, unit: days per year) defined as the number of days per year when grazing is possible with respect to above-ground biomass availability [40], air temperature, snow cover, and soil moisture conditions (see “Specific management strategies in ORCHIDEE-GM v2.2” for detail). The fourth indicator is the beginning of the grazing season (*B*_*grazing*_, unit: day of year) defined in each grid-cell as the first day of the year when the following two criteria are met: (1) grazing is allowed over grassland by model, and (2) grazing will last more than 20 days in the following 30 days. The second criterion is to guarantee that the *B*_*grazing*_ indicates the start of a continual grazing season rather than the sporadic grazing.

### Simulation set-up

ORCHIDEE-GM v2.2 is integrated over Europe using the harmonized climate dataset from the ERA-WATCH reanalysis at a spatial resolution of 0.25° by 0.25° for the period 1901–2010 [49]. A harmonized climate forcing and dynamical downscaling was used for future climate simulations based on the general circulation model (GCM) ECHAM5 (2010–2100) using 1970–2010 as the reference period to correct the GCM bias. Namely, results of the regional climate model (RCM) REMO driven by ECHAM5 GCM climate for the SRES A1B scenario provided by the ENSEMBLE project (http://www.ensembles-eu.org/) are bias-corrected [49] and used in this study. This forcing is described in Beer et al. [49], and hereafter referred to as bias-corrected REMO + ECHAM5 climate. The SRES A1B scenario has a rapid increase in fossil CO_2_ emissions until 2050 and a decrease afterwards, and the projected atmospheric CO_2_ concentration reaches 527.9 ppm in 2050 and 713.8 ppm in 2100. Climate change from ECHAM5 under this scenario leads to a GMST increase comparable to that of RCP6, or between RCP4.5 and RCP8.5 [50]. All climate data have the same spatial resolution of 0.25° by 0.25° and daily temporal resolution. This resolution (0.25° by 0.25°) is sufficient to represent regional meteorological regimes accurately in low-lying regions, but not in mountainous areas. Generally, the patterns of temperature and precipitation change in Europe projected by bias-corrected REMO + ECHAM5 climate (Additional file 1: Figures S1a, b and S2a, b) and non bias-corrected climate (Additional file 1: Figures S1c, d and S2c, d) are similar to those of the ensemble-mean from other RCMs and GCMs in the ENSEMBLES project for the same SRES A1B scenario (Additional file 1: Figures S1e, f and S2e, f; [8]. The changes in ensemble-mean temperature (from the ENSEMBLES project for the SRES A1B scenario) are comparable with those from the more recent EURO-CORDEX ensemble of RCMs for the Representative Concentration Pathways RCP4.5 and RCP8.5 [8]. Global atmospheric CO_2_ concentration used to force ORCHIDEE-GM v2.2 is prescribed from the combination of ice core records and atmospheric observations for 1901–2010 [51] and update) and the SRES A1B scenario for 2011–2100.

The historical simulation for the period 1901–2010 (hereafter referred to as *E*_*hist*_) is exactly the same than the one performed by Chang et al. [12]; see their Fig. 1. For the future period 2011–2100, three experiments are carried out to (1) separate the effect of rising CO_2_ concentration and future climate change, and (2) to investigate the impact of fixed vs. adjustable management on the carbon balance of grasslands. Table 1 presents the drivers for different experiments.

**Fig. 1:**
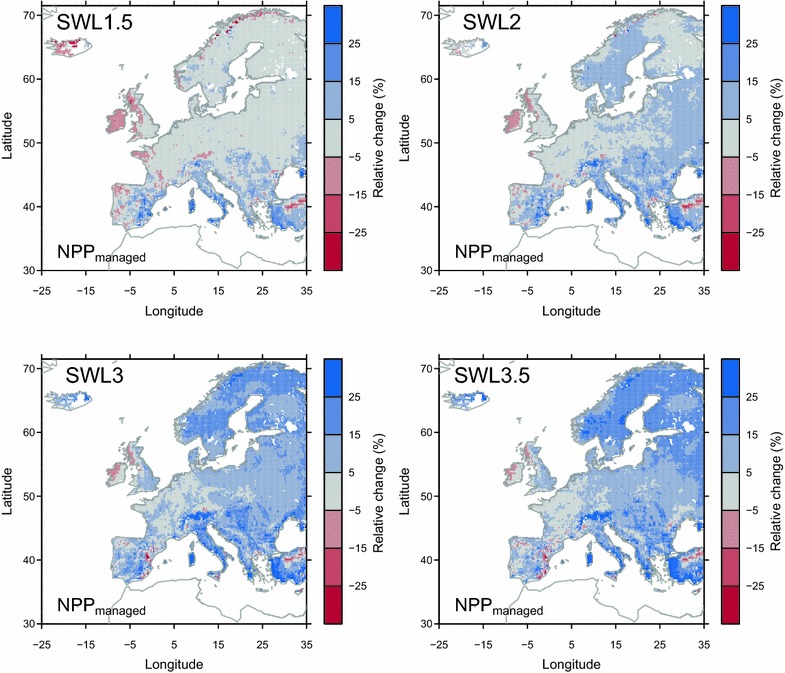
Simulated relative changes of *NPP*_*managed*_ from experiment *E*_*control*_. The change of NPP is defined as the difference between *NPP*_*managed*_ during 30 years in the future corresponding to each SWL minus *NPP*_*managed*_ in reference historical period (1981–2010)

**Table 1:** Drivers for different experiments in this study

Experiment	Climate^a^	Land cover^b^	Managed grassland area^c^	Atmospheric CO_2_ concentration	Management strategy
*E*_*hist*_^d^	Varied for 1901–2010	Varied for 1901–2010	Varied for 1901–2010	Varied for 1901–2010	Adaptive management change algorithm simulating potential livestock density
*E*_*control*_	Varied for 2011–2100	Constant as in 2010	Constant as in 2010	Varied for 2011–2100 (A1B scenario)	Adaptive management change algorithm simulating potential livestock density
*E*_*noco2*_	Varied for 2011–2100	Constant as in 2010	Constant as in 2010	Constant as in 2010	Adaptive management change algorithm simulating potential livestock density
*E*_*fixD*_	Varied for 2011–2100	Constant as in 2010	Constant as in 2010	Varied for 2011–2100 (A1B scenario)	Adaptive management change algorithm with constant livestock density

^a^Climate change for 2011–2100 is predicted by REMO driven by ECHAM5 climate for A1B scenario [49] provided by the ENSEMBLE project (http://www.ensembles-eu.org/)

^b^Land cover is derived from HILDA dataset [52]

^c^Area of managed grassland is calculated using the same method as in previous study for historical period [40]

^d^See Chang et al. [40] for detail protocol of simulation

The first experiment (hereafter referred to as *E*_*control*_) is carried out with increasing atmospheric CO_2_ concentration, and variable climate (bias-corrected REMO + ECHAM5 climate), considering no changes in the nitrogen status of plants (i.e., the same nitrogen effect on photosynthesis as that of 2010; see [12] for detail) and no land cover change (*HILDA* data set; [52]. In more details, a constant land cover map for the period 2011–2100 (Table 1) corresponded to (a) no land-cover change on forest, cropland and grassland area, (b) constant managed/unmanaged grassland fraction as that of 2010 (as calculated in [12], while (c) within managed grassland systems, the intensification/extensification of management (i.e., *D*_*opt*_ increase/decrease), and the fraction between mown and grazed part is determined by the adaptive management change algorithm following the productivity change due to rising atmospheric CO_2_ concentration and variable climate.

The second experiment has the same settings as *E*_*control*_ but uses constant CO_2_ concentration as in the year 2010 (hereafter referred to as *E*_*noco2*_).

The third experiment is like *E*_*control*_ but with constant livestock density over managed grassland as in the year 2010 (thus keeping the same livestock numbers over European grasslands; hereafter referred to as *E*_*fixD*_).

The *E*_*noco2*_ and *E*_*fixD*_ simulations are used in “Discussion” for discussing the effect of rising CO_2_ on productivity and the grassland carbon balance, and the impact of livestock numbers on grassland carbon balance respectively. The results presented in Sect. 3 are all derived from *E*_*hist*_ for the reference period and *E*_*control*_ for the future period.

### Represented time slices

Results for five time slices are presented in this study, namely the reference period (1981–2010) and different SWL of +1.5, +2, +3 and +3.5 °C relative to pre-industrial climate (hereafter referred to as SWL1.5, SWL2, SWL3, and SWL3.5). A SWL is defined as the 30-years period when the average global mean temperature reaches a given warming level compared to the pre-industrial period 1881–1910, following [53]. Due to the fast increase in the projected global mean temperature, there are overlaps across SWL time periods. Table 2 shows the central year and corresponding periods reaching the different SWLs in the ECHAM5 GCM simulation for SRES A1B scenario [54].

**Table 2:** Time period and the corresponding annual mean temperature (*T*_*Europe*_) and annual total precipitation (*P*_*Europe*_) of Europe for which +1.5, +2, +3 and +3.5 °C global warming compared to pre-industrial times was reached in ECHAM5 GCM global climate for SRES A1B scenario provided by the ENSEMBLE project

	SWL^a^ (°C)	*T*_*Europe*_ (°C)	*P*_*Europe*_ (mm year^−1^)	Central year	Period
Reference	+0.68^b^	8.6	818	1995	1981–2010
SWL1.5	+1.5	9.6	818	2035	2021–2050
SWL2	+2	10.2	815	2048	2034–2063
SWL3	+3	11.2	818	2072	2058–2087
SWL3.5	+3.5	11.7	821	2085	2071–2100

^a^ *SWL* specific warming level relative to pre-industrial (1881–1910) climate. A SWL is defined as the 30-years period when the average global mean temperature reaches a given warming level compared to the pre-industrial period 1881–1910, following Vautard et al. [53]. Due to the fast increase in the projected global mean temperature, there are overlaps across SWL time periods

^b^The SWL in ‘Reference’ period (1981–2010) is the average SWL based on the three global observational datasets: NASA GISS (+0.70 °C; http://data.giss.nasa.gov/gistemp/), HadCRUT4 (+0.66 °C; http://www.metoffice.gov.uk/hadobs/hadcrut4/) and NOAA NCDC (+0.68 °C; http://www.ncdc.noaa.gov/cmb-faq/anomalies.html#anomalies). Data were Accessed in September, 2016

Our study domain covers 30 countries (EU-28 plus Norway and Switzerland), which were grouped into a number of major agricultural regions determined by environmental and socio-economic factors (Additional file 1: Table S1, detailed description in [55]. Regional mean biogenic potential productivity (*Y*_*pot*_), grazing capacity (*C*_*grazing*_), optimal livestock density (*D*_*opt*_), and NPP of managed grassland (*NPP*_*managed*_) are averaged based on the managed grassland area in each grid cell. Regional mean net carbon fluxes such as NEE and NBP (calculated as in [12] are averaged based on the total grassland area in each grid cell (including both managed and unmanaged part).

### Factorial attribution of the changes in the grazing season length (*L*_*grazing*_)

In ORCHIDEE-GM v2.2, the grazing is determined by above-ground biomass availability [40] and by air temperature, snow cover, and soil moisture conditions (“Specific management strategies in ORCHIDEE-GM v2.2” for detail). Thus all these four factors could contribute to the simulated changes in *L*_*grazing*_ (i.e., ∆*L*_*grazing*_ between the SWLs and the reference period). To determine the dominant factor affecting changes in grazing season length, ∆*L*_*grazing*_, we define ∆*L*_*grazing, biomass*_, ∆*L*_*grazing, frost*_, ∆*L*_*grazing, snow*_, and ∆*L*_*grazing, wet*_ as the individual contribution from changes on above-ground biomass availability, air temperature, snow cover, and soil moisture conditions respectively on the ∆*L*_*grazing*_. ∆*L*_*grazing, biomass*_ is calculated as:

3 $$\Delta L_{grazing,\;biomass} = L_{grazing,\;biomass,\;SWL} {-}L_{grazing,\;biomass,\;ref}$$

where *L*_*grazing, biomass, SWL*_ and *L*_*grazing, biomass, ref*_ are the mean number of days per year during when grazing is limited by above-ground biomass availability for a given SWL and for the reference period respectively. Here, positive ∆*L*_*grazing, biomass*_ indicates that the changes in above-ground biomass availability tend to lengthen *L*_*grazing*_ (i.e., leading to positive ∆*L*_*grazing*_) and vice versa. ∆*L*_*grazing, frost*_, ∆*L*_*grazing, snow*_, and ∆*L*_*grazing, wet*_ are calculated as:

4 $$\Delta L_{grazing,\;frost} = {-}(L_{grazing,\;frost,\;SWL} {-}L_{grazing,\;frost,\;ref})$$

5 $$\Delta L_{grazing,\;snow} = {-}(L_{grazing,\;snow,\;SWL} {-}L_{grazing,\;snow,\;ref})$$

6 $$\Delta L_{grazing,\;wet} = {-}(L_{grazing,\;wet,\;SWL} {-}L_{grazing,\;wet,\;ref})$$

where *L*_*grazing, frost, SWL*_, (*L*_*grazing, snow, SWL*_, and *L*_*grazing, wet, SWL*_) and *L*_*grazing, frost, ref*_, (*L*_*grazing, snow, ref*_, and *L*_*grazing, wet, ref*_) are the mean number of days per year that grazing is allowed by above-ground biomass availability but prevented by the management strategy to avoid frost-grazing (snow-grazing, and wet-grazing; “Specific management strategies in ORCHIDEE-GM v2.2”) during SWLs and during the reference period respectively. Here, a positive ∆*L*_*grazing, frost*_ value (or ∆*L*_*grazing, snow*_, and ∆*L*_*grazing, wet*_ value) indicates that the changes in the number of freezing days (snow-covering days, and wet soil days) tend to lengthen *L*_*grazing*_ (i.e., leading to a positive ∆*L*_*grazing*_) and vice versa. We defined the *dominant* factor impacting ∆*L*_*grazing*_ as the factor (a) having the same sign of change than ∆*L*_*grazing*_, and (b) having the largest contribution (absolute value) among all the factors.

## Results

### Changes in annual *NPP*_*managed*_, *Y*_*pot*_, *C*_*grazing*_, and *D*_*opt*_ from *E*_*control*_ in response to global change

Compared to the reference period 1981–2010, *NPP*_*managed*_ over European grasslands is projected to change by 0, +4 +8 and +8% under SWL of 1.5, 2, 3 and 3.5 °C respectively, but with large spatial variation (Table 3; Fig. 1). Under SWL of 1.5 °C, we found that (1) *NPP*_*managed*_ decreases by more than 5% over Iceland, western British Isles, Brittany and some regions in south France, and the western Iberian Peninsula, (2) *NPP*_*managed*_ increases by more than 5% over Mediterranean regions and Alps, and (3) little changes in other regions (Fig. 1), compared to the reference period. *NPP*_*managed*_ is simulated to increase with further warming (i.e., under SWL of 2, 3 and 3.5 °C; Fig. 1) over most regions. One exception is the eastern Iberian Peninsula, where *NPP*_*managed*_ decreases under SWL of 3 and 3.5 °C due to a strong decrease in precipitation (Additional file 1: Figure S2b). The spatial pattern of the relative changes in *Y*_*pot*_ follows that of *NPP*_*managed*_, while *Y*_*pot*_ has larger relative increase (Additional file 1: Figure S3). The relative changes in *C*_*grazing*_ show a little different spatial pattern from that of *Y*_*pot*_. An annual *C*_*grazing*_ increase is simulated over most regions of Europe under all SWLs (Additional file 1: Figure S4), including Iceland and the western part of British Isles. And the relative change in annual *C*_*grazing*_ is stronger than that of *Y*_*pot*_ (Table 3). It is noteworthy that consistent decreases in *NPP*_*managed*_, *Y*_*pot*_ and *C*_*grazing*_ were simulated over eastern Iberian Peninsula under SWL of 3 and 3.5 °C. The relative change in *D*_*opt*_ over managed grassland (Additional file 1: Figure S5) is in between changes in *Y*_*pot*_ and in *C*_*grazing*_ (Table 3).

**Table 3:** Simulated productivity of European managed grassland for the reference period (1981–2010), and their relative changes derived from experiment *E*_*control*_

Variables	Period^a^	Average of Europe	Nordic	British Isles	Western	Mediterranean	Alpine	North eastern	South eastern	Eastern
*Y*_*pot*_ (kg DM m^−2^ year^−1^)	Reference	0.85	0.49	0.93	1.07	0.71	0.66	0.80	0.80	0.73
	SWL1.5	6%	6%	−3%	5%	10%	10%	9%	13%	11%
	SWL2	11%	13%	2%	10%	14%	20%	17%	22%	18%
	SWL3	18%	28%	8%	16%	18%	37%	26%	31%	28%
	SWL3.5	20%	36%	11%	18%	16%	46%	29%	33%	35%
*C*_*grazing*_ (LU day ha^−1^)	Reference	208	61	241	286	175	128	187	196	123
	SWL1.5	13%	36%	6%	11%	12%	22%	27%	17%	31%
	SWL2	20%	56%	10%	15%	15%	36%	42%	29%	51%
	SWL3	29%	93%	17%	22%	18%	60%	59%	41%	81%
	SWL3.5	32%	119%	21%	23%	16%	73%	66%	43%	98%
*D*_*opt*_ (LU ha^−1^)	Reference	0.74	0.33	0.84	0.97	0.63	0.51	0.67	0.70	0.54
	SWL1.5	10%	17%	4%	9%	11%	18%	18%	13%	20%
	SWL2	16%	28%	8%	14%	15%	28%	29%	23%	32%
	SWL3	24%	47%	14%	20%	19%	49%	44%	35%	51%
	SWL3.5	28%	61%	17%	23%	20%	59%	50%	39%	61%
*NPP*_*managed*_ (g C m^−2^ year^−1^)	Reference	861	597	937	1035	703	771	867	815	835
	SWL1.5	0%	1%	−7%	−1%	7%	3%	1%	6%	1%
	SWL2	4%	6%	−4%	2%	10%	9%	4%	12%	5%
	SWL3	8%	14%	0%	5%	13%	17%	8%	16%	12%
	SWL3.5	8%	17%	1%	5%	12%	23%	9%	16%	14%

^a^The values for the reference period (1981–2010) are expressed with the original unit, and the relative changes under SWL of 1.5, 2, 3 and 3.5 °C are presented as percent (%) increase (+) or decrease (−) compared to the values for the reference period

**Fig. 2:**
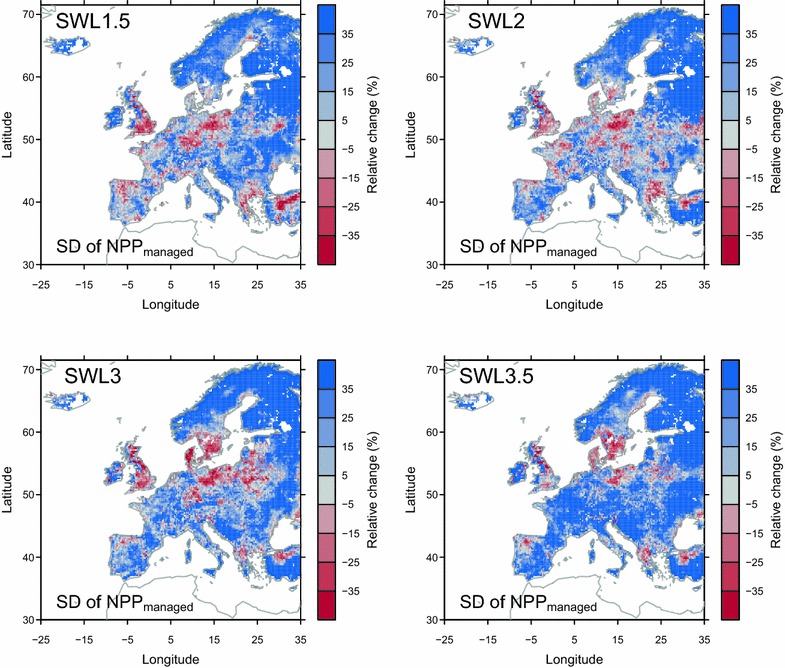
Simulated relative changes in the standard deviation (SD) of *NPP*_*managed*_ from experiment *E*_*control*_

We use the standard deviation (SD) to diagnose the magnitude of the interannual variability in modeled productivity during different time periods. Compared to the reference period, a large increase in SD of *NPP*_*managed*_ is simulated over northern and eastern Europe, Iceland, Ireland, and the western Great Britain under all SWLs (Fig. 2), and over France and most part of the Iberian Peninsula under SWL of 3 and 3.5 °C. Model simulates decrease in SD of *NPP*_*managed*_ over the eastern Great Britain, Denmark, Germany, Poland, northwestern Spain, and northern Greece and southern Sweden under all SWLs.

### Growing season and management shifts

**Fig. 3:**
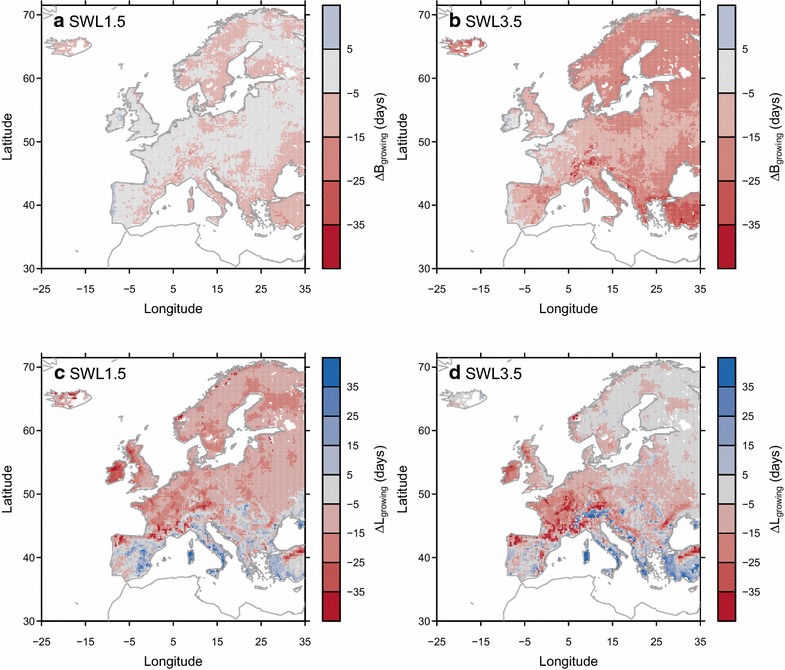
Simulated changes in the start of active growing season (∆*B*_*growing*_; **a**, **b**), and the changes in the length of active growing season (∆*L*_*growing*_; **c**, **d** from experiment *E*_*control*_. Changes are defined as differences between future values and *B*_*growing*_ (**a**, **b**) or *L*_*growing*_ (**c**, **d**) during the reference period (1981–2010) respectively. Negative ∆*B*_*growing*_ indicated an advance in *B*_*growing*_ (i.e., earlier start). Positive ∆*L*_*growing*_ indicated a longer *L*_*growing*_ (i.e., extension of active growing season)

ORCHIDEE-GM v2.2 simulates an earlier start of grassland growing season (i.e., negative ∆*B*_*growing*_ in Fig. 3a, b) over some regions of Europe under SWL of 1.5 °C. The earlier start under SWL of 3.5 °C becomes widespread all over the Europe except in Ireland, western France and eastern Portugal. However, this earlier start of the active growing season does not necessarily translate into a longer active growing season. Increasing *L*_*growing*_ is simulated only over some Mediterranean regions, Alps, and mountains in Eastern Europe under all SWLs, and decreasing *L*_*growing*_ over other regions (Fig. 3c, d).

**Fig. 4:**
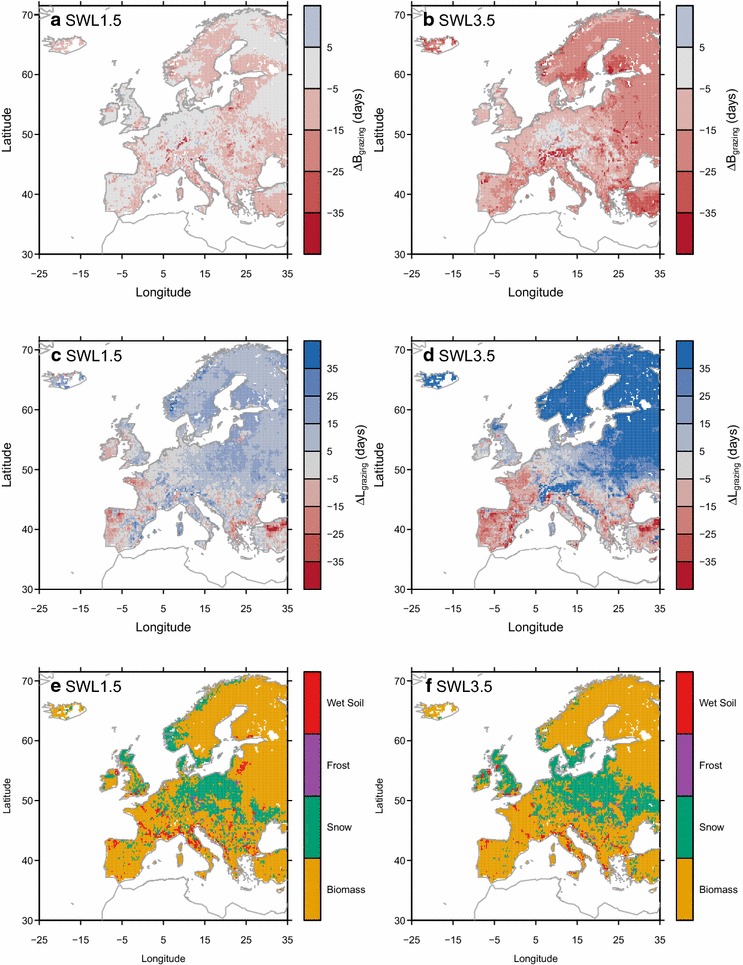
Simulated changes in the start of grazing season (∆*B*_*grazing*_; **a**, **b**), the changes in the length of grazing season (∆*L*_*grazing*_; **c**, **d**) from experiment *E*_*control*_, and the dominant factor impacting the ∆*L*_*grazing*_ for SWL of **e** +1.5 ºC and **f** +3.5 ºC. Changes are defined as differences between future values and *B*_*grazing*_ (**a**, **b**) or *L*_*grazing*_ (**c** and** d**) during the reference period (1981–2010) respectively. Negative ∆*B*_*grazing*_ indicated an advance in *B*_*grazing*_ (i.e., earlier start). Positive ∆*L*_*grazing*_ indicated a longer *L*_*grazing*_ (i.e., extension of grazing season)

An earlier start of the grazing season (i.e., negative ∆*B*_*grazing*_ in Fig. 4a, b) was simulated over some European grassland under SWL of 1.5 °C, and over most part of Europe under SWL of 3.5 °C. The largest advance was simulated over Alps and northern Europe under SWL of 3.5 °C. Longer grazing seasons (i.e., positive ∆*L*_*grazing*_ in Fig. 4c, d) were simulated over most parts of Europe, except in the Atlantic coast (western British Isles, western France, western Spain, and Portugal), southern Italy and Greece under all SWLs. Under SWL of 3.5 °C, shorter grazing seasons were simulated over France and the Iberian Peninsula, as well as in most parts of Italy and southeastern Europe. Attribution analysis of ∆*L*_*grazing*_ (“Factorial attribution of the changes in the grazing season length (*Lgrazing*)”; Fig. 4e, f) shows that (1) above-ground biomass availability is the dominant factor determining ∆*L*_*grazing*_ in most regions of Europe (Additional file 1: Figure S6), (2) snow cover is the dominant factor over some regions of British Isles, the coast of the North Sea and Baltic Sea, and eastern Europe (Additional file 1: Figure S7), (3) the impact of changes in frost days have similar spatial patterns but weaker than the impact of snow cover (Additional file 1: Figure S8), and (4) wet soils only play a dominant role in explaining ∆*L*_*grazing*_ over few regions (Additional file 1: Figure S9). It is noteworthy that snow-impact dominated regions do expand under SWL3.5 in mid-to-high latitude Europeans regions (Fig. 4f), indicating that changes in snow cover become an increasingly important factor impacting *L*_*grazing*_ under higher warming level.

**Fig. 5:**
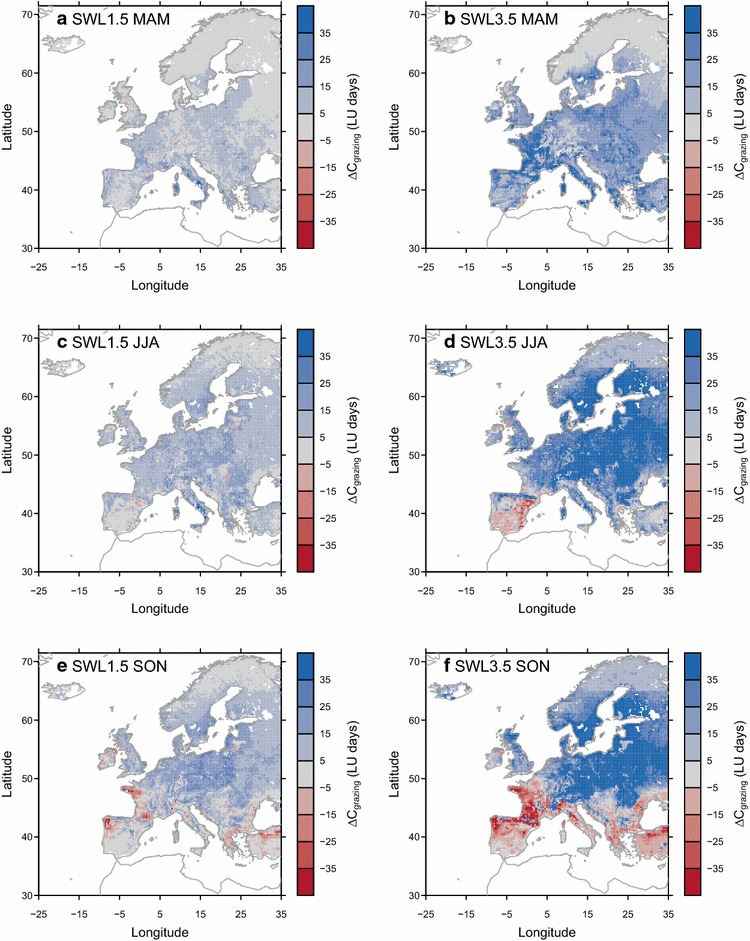
Projected seasonal changes of grazing capacity (*C*_*grazing*_) from experiment *E*_*control*_. Changes are defined as differences between future values and seasonal *C*_*grazing*_ during the reference period (1981–2010) respectively. In this study, we defined seasons in Europe as: March, April and May (MAM) for spring; June, July and August (JJA) for summer; September, October and November (SON) for autumn; December, January and February (DJF) for winter. Due to the fact that few winter grazing could happened in Europe, we only showed the changes of grazing capacity for the other three seasons

Following the simulated changes in grazing season and in *S*_*opt*_, we found a *C*_*grazing*_ increase for spring (March, April and May; Fig. 5a, b) under all SWLs and over most part of Europe except in high-latitude regions where grass leaf onset is usually later than May. European-wide *C*_*grazing*_ increase was also simulated for summer (June, July and August; Fig. 5c) under SWL of 1.5 °C, while summer *C*_*grazing*_ tended to decrease over the southern Iberian Peninsula, and Mediterranean coast of France under SWL of 3.5 °C. For autumn (September, October and November; Fig. 5e, f), a significant *C*_*grazing*_ decrease was simulated over regions where grazing season become shorter in the future (Fig. 4c, d), due to an earlier end to the growing season.

### The carbon balance of European grassland in the future

**Fig. 6:**
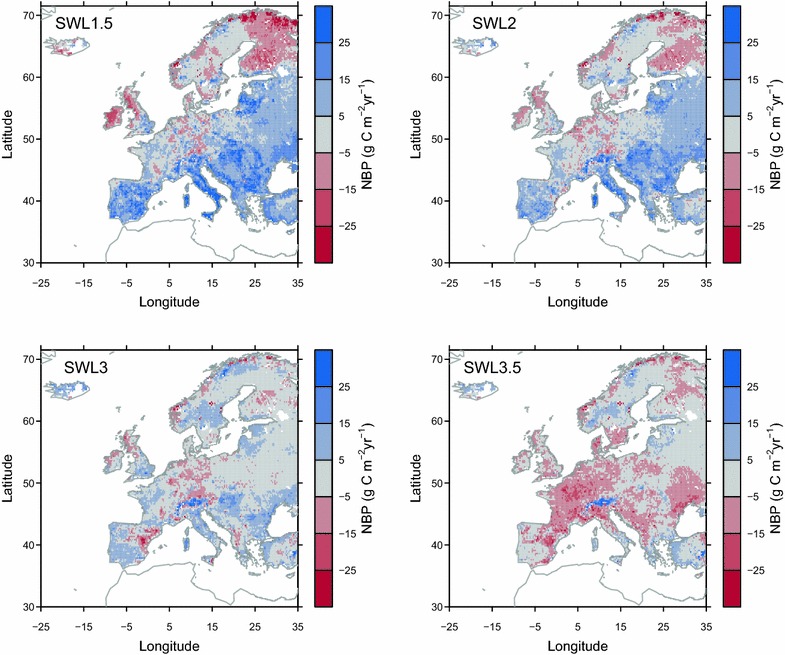
Simulated grassland NBP from experiment *E*_*control*_. Positive NBP indicated a carbon sink

Under SWL of 1.5 °C, ORCHIDEE-GM v2.2 simulates a carbon sink in grassland soils (i.e., positive NBP) over Southern and Eastern Europe, and a carbon source over the western British Isles, and some regions in Western and Northern Europe (Fig. 6). Over most parts of the Europe, NBP was simulated to decrease along further warming (i.e., under SWL 2, 3 and 3.5 °C; Table 4). Under SWL of 3.5 °C, most part of the European grassland was simulated to be carbon neutral (e.g., western Iberian Peninsula, and some regions in northeastern Europe, and northern Europe; Fig. 6) or carbon source. One exception is the Alps, where carbon sink was simulated under all SWLs.

**Table 4:** The NBP of European grassland simulated by ORCHIDEE-GM v2.2 from experiment *E*_*control*_, *E*_*fixD*_, and *E*_*noco2*_

Experiment	Period	Europe	Nordic	British Isles	Western	Mediterranean	Alpine	North eastern	South eastern	Eastern
*E*_*control*_	SWL1.5	6 ± 17	−3 ± 31	−10 ± 23	1 ± 23	17 ± 46	10 ± 26	8 ± 23	19 ± 57	16 ± 35
	SWL2	5 ± 17	−1 ± 32	−5 ± 20	−0 ± 23	11 ± 61	11 ± 23	5 ± 24	17 ± 45	13 ± 36
	SWL3	2 ± 24	2 ± 36	−2 ± 23	−1 ± 32	3 ± 51	9 ± 21	0 ± 21	7 ± 64	6 ± 26
	SWL3.5	−3 ± 32	−0 ± 33	−5 ± 25	−9 ± 49	−3 ± 59	10 ± 20	−4 ± 26	−4 ± 83	4 ± 28
*E*_*fixD*_	SWL1.5	7 ± 17	−2 ± 31	−10 ± 23	1 ± 23	18 ± 46	11 ± 26	10 ± 23	19 ± 57	17 ± 35
	SWL2	6 ± 17	0 ± 32	−4 ± 20	0 ± 23	13 ± 61	14 ± 23	8 ± 24	19 ± 45	14 ± 36
	SWL3	4 ± 25	4 ± 37	−0 ± 23	0 ± 32	5 ± 52	14 ± 21	4 ± 20	10 ± 64	8 ± 26
	SWL3.5	−2 ± 32	2 ± 34	−2 ± 24	−8 ± 49	−3 ± 60	15 ± 19	−0 ± 26	−2 ± 83	6 ± 29
*E*_*noco2*_	SWL1.5	−12 ± 16	−14 ± 27	−27 ± 20	−20 ± 24	−4 ± 42	−5 ± 21	−7 ± 23	−2 ± 58	−1 ± 31
	SWL2	−17 ± 16	−15 ± 27	−27 ± 22	−25 ± 26	−14 ± 54	−8 ± 19	−13 ± 22	−7 ± 47	−7 ± 30
	SWL3	−17 ± 22	−13 ± 26	−19 ± 23	−22 ± 36	−19 ± 42	−9 ± 15	−17 ± 25	−15 ± 64	−14 ± 22
	SWL3.5	−21 ± 30	−14 ± 22	−19 ± 25	−28 ± 55	−22 ± 46	−9 ± 16	−21 ± 34	−24 ± 81	−14 ± 19

The NBP is presented as 30-year mean value ± standard deviation of interannual variability. Positive value indicates a carbon sink, while negative value indicates a carbon source of grassland ecosystem

## Discussion

### Changes in productivity and phenology of European grassland under future global change: mechanisms and implications for grassland management

**Fig. 7:**
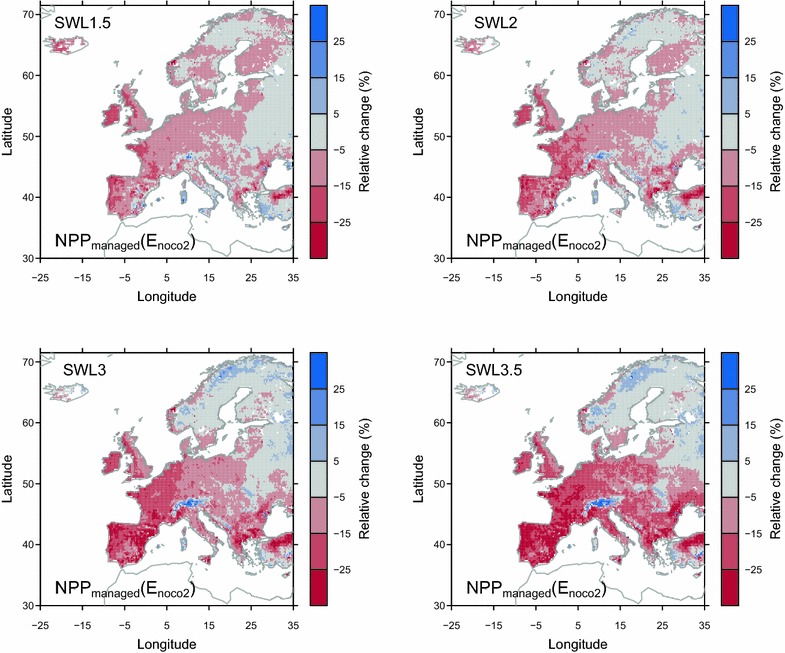
Simulated relative changes of *NPP*_*managed*_ from experiment *E*_*noco2*_

The increase in productivity over European grasslands under SRES A1B REMO + ECHAM5 bias-corrected climate and atmospheric CO_2_ concentration prescribed to ORCHIDEE-GM v2.2 is mainly due to the effect of rising CO_2_ concentration. In fact, the NPP increase is diminished (e.g., in some region of high-latitude Scandinavia, and in Alps) or NPP even decreases when excluding the effect of rising CO_2_ (*E*_*noco2*_; Fig. 7). Elevated CO_2_ concentration has the dual effect of increasing leaf photosynthesis, leaf area index, and reducing stomatal conductance, thus increasing water-use efficiency [17] and indirectly reducing the consumption of soil moisture by transpiration [18]. An increase in productivity induced by elevated CO_2_ has been documented in several grassland FACE experiments (e.g., [15, 56–58]), while actual productivity changes were impacted by interactions among the elevated CO_2_ concentration, climate [59], soil conditions [60], nitrogen availability [61–64], and community composition [57, 65]. In this study, ORCHIDEE-GM v2.2 does not include nitrogen cycling, and we assumed a constant nitrogen status of grass in the future as that of 2010. Thus the projected productivity is only modulated by the effects of CO_2_ and climate, but not by nitrogen availability. In the reality, changes in NPP will also be strongly modulated by the availability of soil nutrients [61] and their recycling by grazing animals. In particular, legumes could override CO_2_ induced nitrogen limitation by increasing biological nitrogen fixation in grasslands [62, 66]. Besides affecting biomass production (yield), climate change can also impact the nutritional value of grass feed [67] through changing nutrition content of individual species and species composition [68], and altering the optimal timing and number of harvest (e.g., in northern Europe; [69]. The importance of nutrient effect on grassland productivity and forage quality represents a priority of including nutrient cycling (e.g., nitrogen and phosphorus) in ORCHIDEE-GM v2.2 in the future. Furthermore, grassland in different regions may have specific response to climate change, which brings challenges for grassland modeling [67]. For example, perennial grassland sward performance (e.g., productivity) may be affected by winter conditions (e.g., extreme cold events or snow cover depth; [70, 71], and Mediterranean grasslands with dominant annual species could have specific response to climate change different from perennial species [67]. However, more experiment data is required for model development to account for these specific responses [67].

In addition to the changes in annual productivity, we also project a significant shift in grasslands phenology under future climate change, with an earlier spring onset of growth especially under high warming levels (Fig. 3a, b) and changes in growing season length (Fig. 3c, d). Trends towards earlier spring onset of growth were also documented by a number of studies (e.g., [72–75]) which could result from the advanced snowmelt date and warmer temperatures in subalpine and high latitude grassland ecosystems. However, the projected increase in winter precipitation across the mid- and high-latitude regions [2] makes the timing of modeled snowmelt, and hence future projections of phenological shifts, uncertain [76]. For European ecosystems, Menzel et al. [77] documented a general advance in spring leafing during the period 1971–2000. In Mediterranean shrub- and grasslands, advancement in leaf-out events was also observed during the recent decades since the 1970s [78, 79]. According to the projections by ORCHIDEE-GM v2.2, the earlier winter-spring onset will continue over most European grasslands (Fig. 3a, b).

One important result of this study is that the projected increase of summer drying and rising temperatures in Mediterranean regions (IPCC [2]) will probably result in a shift of the active season, with an earlier onset of winter-spring growth being (at least partially) offset by an earlier, and longer lasting, summer drought period. An earlier end to the growing season is predicted in this study over large part of Europe, especially in Western Europe and northern Spain (Fig. 3). This earlier end to the growing season and thus the lower productivity (either decrease or less increase in productivity in the future; Fig. 1) are related to the reduction of mid-summer soil moisture in the model, given the high positive correlation (p < 0.01) between detrended annual NPP variations and summer soil moisture variation over these regions (Additional file 1: Table S2). Our results are consistent with the findings from the simulation of 12 contrasted French grassland sites using PaSim, which show higher spring forage production, but a increasing risks of severe summer shortfalls in the future. The reduction in soil moisture (averaged over 0–9 cm in depth; Additional file 1: Figure S10) is not attributed to individual drivers in the model, but it reflects the combination of continued warming and the reduction in precipitation during summer and autumn from bias-corrected REMO + ECHAM5 climate, and possibly the fact that earlier leaf onset and warmer spring accelerates soil water depletion and thus increases water stress later during the season. Field experiments also suggest that warming and associated soil drying could reduce primary production in many temperate grasslands [80, 81].

With respect to grassland management in the future, the simulated general increase in annual grassland productivity (i.e., NPP) generally leads to an increase in the annual forage yield (*Y*_*pot*_), annual grazing capacity (*C*_*grazing*_), and thus of potential livestock density (*D*_*opt*_), when management is allowed in the model to continually adjust to increasing NPP (i.e., *E*_*control*_). However, there are some practical issues should be noted in this management intensification potential driven by the projected productivity increase. While the trampling effect on growth during grazing has been considered in our model [35]; derived from Vuichard et al. [38], the impact of intensive grazing on soil physics (e.g., soil compaction) and its consequence on soil hydrological and thermal properties [67] is not taken into account and should be addressed by models in the future. In addition, increasing livestock density might bring animal health problem in practice [67] due to various factors such as pathogen spread (e.g., [82]), parasite load, and exposure to environmental extremes.

Our results suggest that the phenological shifts in the future will enable an earlier start of grazing over European grasslands (Fig. 4a, b), which potentially should reduce costs of managing livestock indoors and reduce GHG emissions caused by manure management. We also project an extension of the grazing season over the Alps, and the north and east part of Europe (i.e., positive ∆*L*_*grazing*_, in Fig. 4c, d), even for the regions where the active growing season shortens in the future (Fig. 4c, d). The reason of this decoupling between the duration of growing vs. grazing season could possibly be attributed (Fig. 4e, f) to (1) the enhanced productivity (i.e., NPP) during growing season, which allows a longer grazing season, (2) the later snow cover (Additional file 1: Figure S7), (3) less days with daily mean air temperature drops below 0 °C (Additional file 1: Figure S8), and (4) reduction in waterlogging events (Additional file 1: Figure S9). In addition, the simulated shift in seasonal and annual grazing capacity (*C*_*grazing*_; Fig. 5; Additional file 1: Figure S4) suggests that under future warming, 1) over most regions in Europe, more livestock could be put over pasture during growing season (Fig. 5a, b), (2) an earlier grazing is enabled by the advance of leaf onset (Fig. 4a, b), (3) for regions such as northern Spain and France, less livestock could be supported by grassland during summer and/or autumn (Fig. 5d–f), thus causing an increasing needs of conserved forage for summer and/or autumn utilization by ruminant livestock [27].

**Fig. 8:**
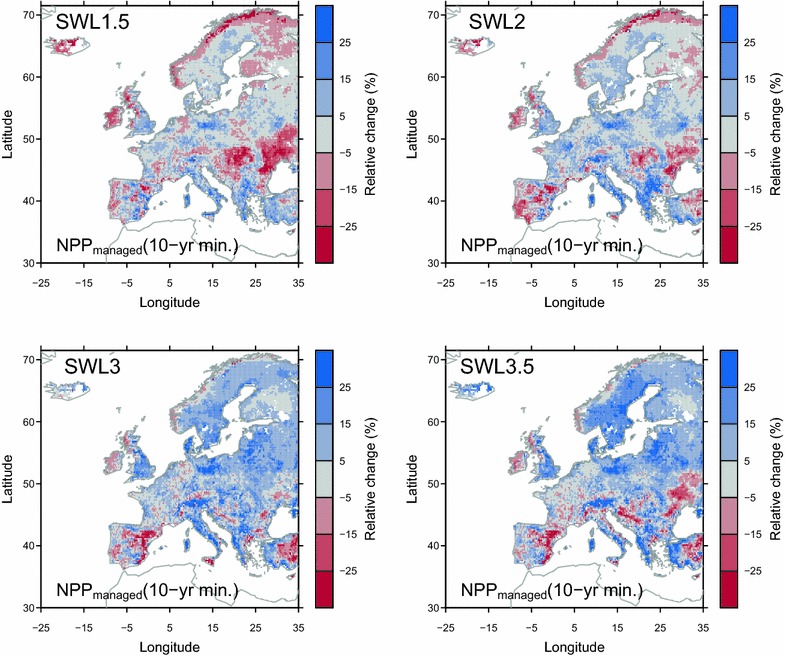
Simulated relative changes of 10-year minimum *NPP*_*managed*_ from experiment *E*_*control*_. The 10-year minimum *NPP*_*managed*_ is defined as minimum *NPP*_*managed*_ in the past 10 years. The change of 10-year minimum *NPP*_*managed*_ is defined as the the difference between 10-year minimum *NPP*_*managed*_ during 30 years in the future corresponding to each SWL minus 10-year minimum *NPP*_*managed*_ in reference historical period (1981–2010)

Given the coincident increase in the interannual variability of NPP and the mean value of NPP over some regions (e.g., northern Europe; Fig. 2), farmers may not be able to take a full advantage of the long-term mean productivity increase, or may even be negatively affected by larger productivity variations. In practice, farmers end to adopt a lower livestock density than the potential mean to avoid the risk of insufficient grass for livestock, since in the case of a bad year with low NPP, supplementary feeding is required (e.g., hay and silage) which increases the costs. To assess the possible impact of interannual variability in productivity on future management change, we assumed that farmers adapt their livestock density to the simulated minimum productivity in the past 10 years (i.e., 10-year minimum *NPP*_*managed*_). In most regions of Europe, a weaker increase (or a stronger decrease) of 10-year minimum *NPP*_*managed*_ (Fig. 8) than the increase (or the decrease) of mean *NPP*_*managed*_ (Fig. 1) was found. However, stronger increase in 10-year minimum *NPP*_*managed*_ than that in mean *NPP*_*managed*_ was found over southeastern United Kingdom, Denmark, and some regions of Germany and Poland (Fig. 8), where strong decrease in SD of *NPP*_*managed*_ was simulated (Fig. 2). Similar changes were found for simulated *Y*_*pot*_ (Additional file 1: Figure S11) and *C*_*grazing*_ (Additional file 1: Figure S12), if 10-year minimum *Y*_*pot*_ and *C*_*grazing*_ were adopted by farmers rather than mean value. The above findings suggest that if a conservative adaptation strategy is adopted (i.e., farmers adjust livestock density based on the lowest productivity in the past decade), farmers who confronted to the increased interannual variability in grassland production may, in the future, benefit less from the productivity increase or suffer more losses from the productivity decrease. Adopting a moderate increase in stocking density may reduce the vulnerability of livestock production systems to increased climate variability. Yet, an alternative adaptation strategy (not considered here) would also consist in increasing forage (hay, silage) stocks to reduce risks of low NPP available for grazing.

### Implications for grassland CO_2_ fluxes

ORCHIDEE-GM v2.1 simulated a carbon sink (i.e., a positive NBP of 27 ± 8 g C m^2^ year^2^) over European grassland in the past decade (i.e., 2001–2010; [12]. In this study, we project with ORCHIDEE-GM v2.2 a persistence of the grassland carbon sink over Mediterranean, Alpine, North eastern, South eastern and Eastern regions under SWL of 1.5 and 2 °C (Table 4), associated with longer growing seasons (Fig. 3), and with higher productivity from elevated CO_2_ (Fig. 1). In our simulation, elevated CO_2_ concentration made the dominant contribution to the projected sustained carbon sink, given the fact that NBP becomes a carbon source when the effect of CO_2_ is excluded (NBP from *E*_*noco2*_ in Table 4), which is consistent with the findings from French grassland sites [27]. Both the amount [83] and interannual variability of NEE [84, 85] are sensitive to growing season length, with generally greater net C uptake at sites with a longer, rather than shorter growing season [86]. The extended growing season in Mediterranean, Alpine, and South-eastern regions (Fig. 3) likely drive the simulated carbon sink over those regions in our study. However, this carbon sink is modeled to be much weaker than that in the past, even with the beneficial effects of increasing CO_2_, and the sinks gradually turns to carbon source with further climate change for SWL reaching above 3 °C (Fig. 6; Table 4). For British Isles and Western region, shorter future growing season (Fig. 3) could also contribute to the shift towards a carbon source. For France, slightly positive or neutral NBP was simulated under SWL of 1.5 °C to SWL of 3 °C, consistent with the findings from the detailed French sites simulations [27]. Increase in Rh under future warming (Additional file 1: Figure S13) largely offsets the C gain from the extension of growing season and elevated CO_2_, resulting in most European grasslands becoming a carbon source under SWL of 3.5 °C (Fig. 6). In addition to changes in long-term grassland soil carbon status, there could be risks of grassland degradation and loss of carbon sink during climate extremes such as heat waves and drought events. For example, a probabilistic risk analysis based on simulations with the PaSim model showed increased drought risk in the future for grassland productivity in Southern Europe [28]. However, a series of experiments showed that annual C balance is preserved under elevated CO_2_ despite the extreme [20] and that grasslands productivity decreased under drought event [87, 88] but showed some resilience under elevated CO_2_ [87].

**Fig. 9:**
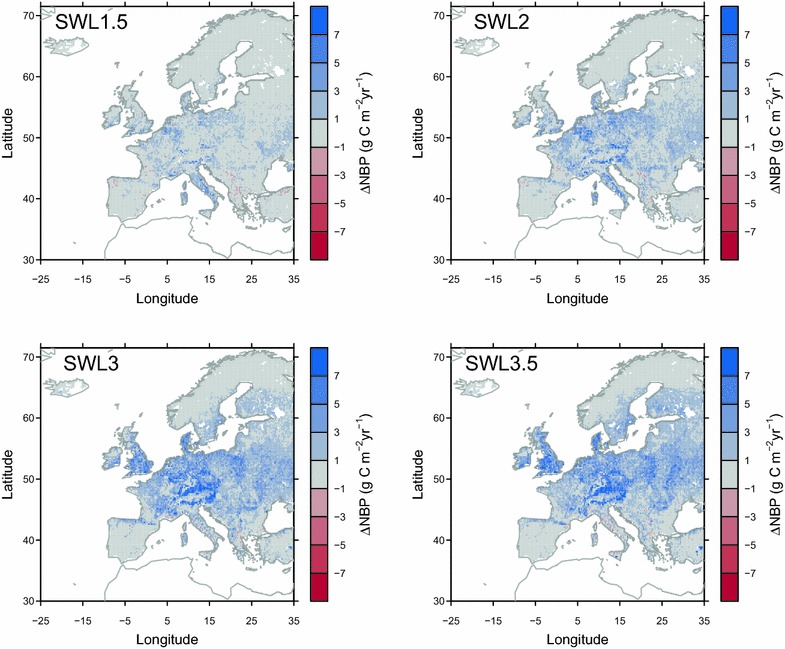
The difference between simulated grassland NBP from experiment *E*_*fixD*_ and that from experiment *E*_*control*_. Positive difference indicates that a lower management intensity either enhances the carbon sink or alleviates the appearance of carbon source

It should be noted that the future carbon balance of European grassland is projected in this study under the assumption of management practice is determined by our adaptive management change algorithm which gives a potential livestock density (*D*_*opt*_) within the managed grassland (“Simulation set-up”). Under this assumption, we obtain an increase in livestock numbers sustained by European managed grassland by 9, 15, 24, and 29% under SWL of 1.5, 2, 3 and 3.5 °C respectively, which means an intensification of grassland management. To assess the impact of adapting management intensity on the carbon balance of European grassland, we look at the results of the sensitivity experiment with constant livestock numbers at the value of the year 2010 (i.e., *E*_*fixD*_). In *E*_*fixD*_, the lower management intensity either enhances the carbon sink or alleviates the appearance of carbon source in the future (Fig. 9; Table 4) because of a lower carbon export from grassland ecosystem (i.e., a larger return of NPP to soils). The effect of the lower management intensity is mainly found over mid-latitude Europe (Fig. 9), and stronger effect can be found under higher SWL due to a larger difference in management intensity between *E*_*control*_ and *E*_*fixD*_. The simulated NBP change through lowering livestock density suggests that there is a possibility of managing (increasing) the carbon sink of European grassland in the future by maintaining/lowering current stocking densities, letting higher NPP under moderate climate change increase soil carbon storage.

An enhanced carbon sink might also be achieved by ameliorating the nutrient status of plants [61]. This could be implemented by additional mineral fertilization, more manure, and introducing/increasing legumes [62, 66]. However, additional fertilization (mineral or manure) may increase the N_2_O emission offsetting the carbon sink, as well as nitrogen leaching losses.

The modeled intensification in grassland management affects the carbon balance of European grassland, and should also impact CH_4_ and N_2_O emissions. Enteric fermentation CH_4_ emissions generally follow the livestock density (e.g., [12]. The simulation of the nitrogen related fluxes such as N_2_O emission requires an explicit nitrogen cycle interacting with the carbon cycle (e.g., [89], which is not included in the current version of ORCHIDEE-GM v2.2. Moreover, changes in livestock density could alter the associated nitrogen cycle during grazing, which potentially impact both the carbon balances and the N_2_O emissions.

## Conclusions

Using the ORCHIDEE-GM v2.2 managed grassland model, increase in grassland production was simulated in response to future global change over Europe, which is mainly due to the effect of rising CO_2_ concentration. The model projects shifts in phenology characterized by an earlier winter-spring onset of grass growth over Europe caused by warming. A longer growing season is only projected over some regions in southern and southeastern Europe. In other regions, an earlier summer-autumn leaf fall caused an even shorter growing season, which could be related to the increased summer drying (i.e., lower soil moisture). The productivity increase and phenological shift enable an increase in potential management intensity with higher annual forage yield, grazing capacity and potential livestock density over European grassland, and a shift in seasonal grazing capacity. The model also projects a continual grassland carbon sink under SWL of 1.5 and 2 °C in most regions of Europe (Mediterranean, Alpine, North eastern, South eastern and Eastern regions). However, a reduced carbon sink or enhanced carbon source is projected over European grassland under continual warming. It should be kept in mind that the results obtained in this study are based on the climate projection from a single climate model. The changes in grassland productivity and phenology might depend on the choice of this specific climate model.

## Additional file

**Additional file 1.** Additional tables and Additional figures.

## Abbreviations

DM: dry matter
GCM: general circulation model
GHG: greenhouse gas
GMST: global mean surface temperature
LU: livestock unit
NBP: net biome productivity
NEE: net ecosystem exchange
NPP: net primary productivity
RCP: representative concentration pathways
Rh: heterotrophic respiration
SRES: special report on emission scenario
SWL: specific warming levels
UNFCCC: United Nations Framework Convention on Climate Change
