# Supplementary material for: Future productivity and phenology changes in European grasslands for different warming levels: implications for grassland management and carbon balance
Source: Carbon Balance Manag. 2017 May 4;12:11. doi: 10.1186/s13021-017-0079-8 (PMC5418182; doi:10.1186/s13021-017-0079-8)
Supplement: Supplementary file 1 — Additional file 1. Additional tables and Additional figures. [file 13021_2017_79_MOESM1_ESM.docx]

**Additional Material**

Table S1 Major agricultural regions in Europe (Olesen & Bindi, 2002).

| Regions | Countries |
| --- | --- |
|  |  |
| Nordic | Norway, Sweden, and Finland |
| British Isles | Ireland, and United Kingdom |
| Western | Denmark, Germany, Netherlands, Belgium, Luxembourg, and France |
| Mediterranean | Portugal, Spain, Italy, Greece, Malta, and Cyprus |
| Alpine | Switzerland, and Austria |
| North eastern | Poland, Czech Republic, and Slovakia |
| South eastern | Hungary, Slovenia, Croatia, Romania, and Bulgaria |
| Eastern | Estonia, Latvia, and Lithuania |

Table S2. Correlation between detrended annual NPP variation (*NPP_IAV_*) and spring temperature variation (*T_springIAV_*), and detrended *NPP_IAV_* and summer soil moisture (average over 0-10cm in depth) variation (*mc_summerIAV_*). In Europe, spring include March, April and May, and summer include June, July and August. The test statistic is based on Pearson's product moment correlation coefficient. Significant correlation with p value < 0.05 is annotated with *, and with p value < 0.01 is annotated with **.

|  | Period | Europe | Nordic | British Isles | Western | Mediterranean | Alpine | North eastern | South eastern | Eastern |
| --- | --- | --- | --- | --- | --- | --- | --- | --- | --- | --- |
| *NPP_IAV_* vs. *T_springIAV_* | Reference | 0.43* | 0.63** | 0.70** | 0.51** | -0.16 | 0.81** | 0.53** | 0.04 | 0.5** |
|  | SWL1.5 | 0.52** | 0.64** | 0.60** | 0.55** | -0.48** | 0.53** | 0.68** | 0.14 | 0.78** |
|  | SWL2 | 0.16 | 0.64** | 0.43* | 0.46* | -0.62** | 0.7** | 0.8** | -0.11 | 0.81** |
|  | SWL3 | 0.17 | 0.46** | 0.57** | 0.51** | -0.51** | 0.61** | 0.76** | -0.28 | 0.56** |
|  | SWL3.5 | -0.08 | 0.39* | 0.48** | -0.1 | -0.69** | 0.71** | 0.65** | -0.34 | 0.65** |
| *NPP_IAV_* vs. *mc_summerIAV_* | Reference | 0.58** | 0.00 | 0.72* | 0.68** | 0.87** | -0.44* | 0.43* | 0.94** | 0.72** |
|  | SWL1.5 | 0.42* | -0.37* | -0.24 | 0.19 | 0.90** | -0.48** | 0.28 | 0.92** | -0.36 |
|  | SWL2 | 0.29 | -0.32 | -0.02 | 0.22 | 0.92** | -0.33 | 0.31 | 0.92** | -0.31 |
|  | SWL3 | 0.62** | -0.53** | -0.12 | 0.63** | 0.89** | -0.37* | -0.06 | 0.90** | -0.45* |
|  | SWL3.5 | 0.66** | -0.68** | -0.36 | 0.8** | 0.92** | -0.53** | 0.27 | 0.91** | -0.36 |


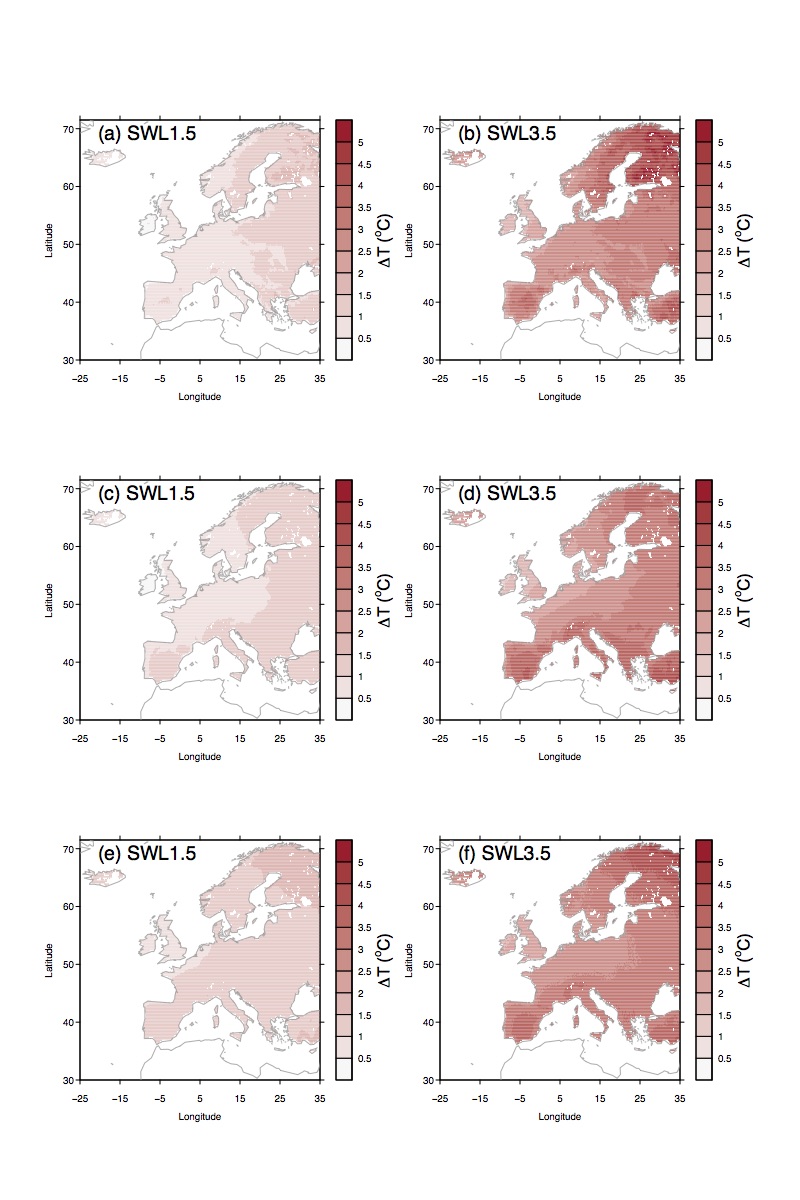


Figure S1. Simulated changes of annual mean temperature (ºC) for SWL of +1.5 ºC, and +3.5 ºC compared to 1981-2010 derived from bias-corrected bias-corrected REMO + ECHAM5 climate for SRES A1B scenario (a and b), from non bias-corrected REMO + ECHAM5 climate for SRES A1B scenario provided by the ENSEMBLE project (c and d), and from ensemble-mean of the RCM+GCM combinations for SRES A1B scenario provided by the ENSEMBLE project (e and f). Totally, 18 RCM+GCM combinations covering the period 1951-2100 were used for the ensemble-mean climate, which are available at: <http://ensemblesrt3.dmi.dk/>. They are (expressed as RCM+GCM) C4IRCA3+HadCM3Q16, CNRM-RM5.1+ARPEGE, HIRHAM5+ECHAM5, HIRHAM5+ECHAM5, HIRHAM5+BCM, CLM+HadCM3Q0, RACMO2+ECHAM5, HadRM3Q0+HadCM3Q0, HadRM3Q16+HadCM3Q16, HadRM3Q3+HadCM3Q3, REMO+ECHAM5, RCA+BCM, RCA+ECHAM5, RCA+HadCM3Q3, RACMO2+ECHAM5-r1, RACMO2+ECHAM5-r2, RACMO2+MIROC3.2, and RACMO2+ECHAM5-r3 (50km resolution).


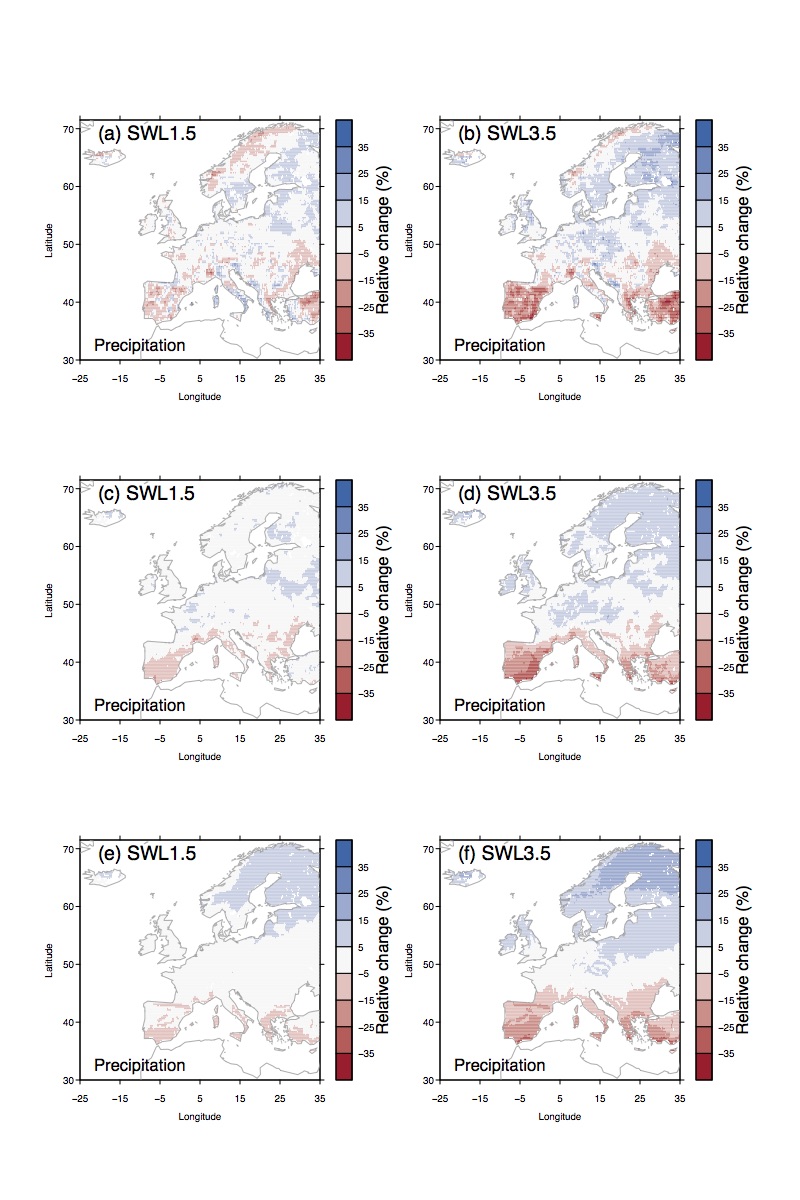


Figure S2. Simulated relative changes of total annual precipitation (%) for SWL of +1.5 ºC, and +3.5 ºC compared to 1981-2010 derived from bias-corrected bias-corrected REMO + ECHAM5 climate for SRES A1B scenario (a and b), from non bias-corrected REMO + ECHAM5 climate for SRES A1B scenario provided by the ENSEMBLE project (c and d), and from ensemble-mean of the RCM+GCM combinations for SRES A1B scenario provided by the ENSEMBLE project (e and f). Totally, 18 RCM+GCM combinations covering the period 1951-2100 were used for the ensemble-mean climate, which are available at: <http://ensemblesrt3.dmi.dk/>. They are (expressed as RCM+GCM) C4IRCA3+HadCM3Q16, CNRM-RM5.1+ARPEGE, HIRHAM5+ECHAM5, HIRHAM5+ECHAM5, HIRHAM5+BCM, CLM+HadCM3Q0, RACMO2+ECHAM5, HadRM3Q0+HadCM3Q0, HadRM3Q16+HadCM3Q16, HadRM3Q3+HadCM3Q3, REMO+ECHAM5, RCA+BCM, RCA+ECHAM5, RCA+HadCM3Q3, RACMO2+ECHAM5-r1, RACMO2+ECHAM5-r2, RACMO2+MIROC3.2, and RACMO2+ECHAM5-r3 (50km resolution).


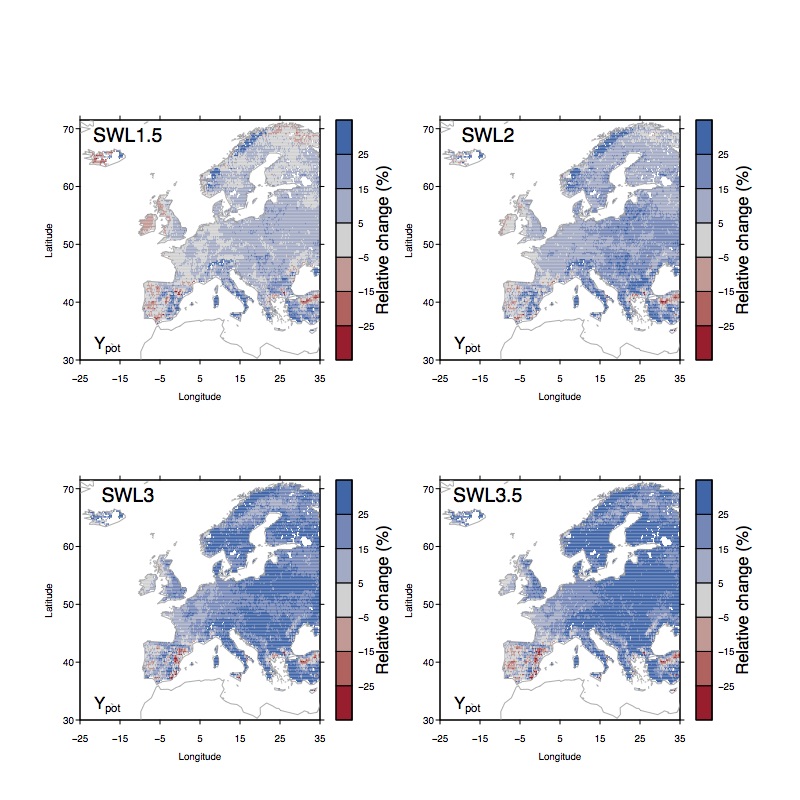


Figure S3. Simulated relative changes of biological potential productivity (*Y_pot_*) from experiment *E_control_*. The change of *Y_pot_* is defined as the difference between *Y_pot_* during 30 years in the future corresponding to each SWL minus *Y_pot_* in reference historical period (1981-2010).


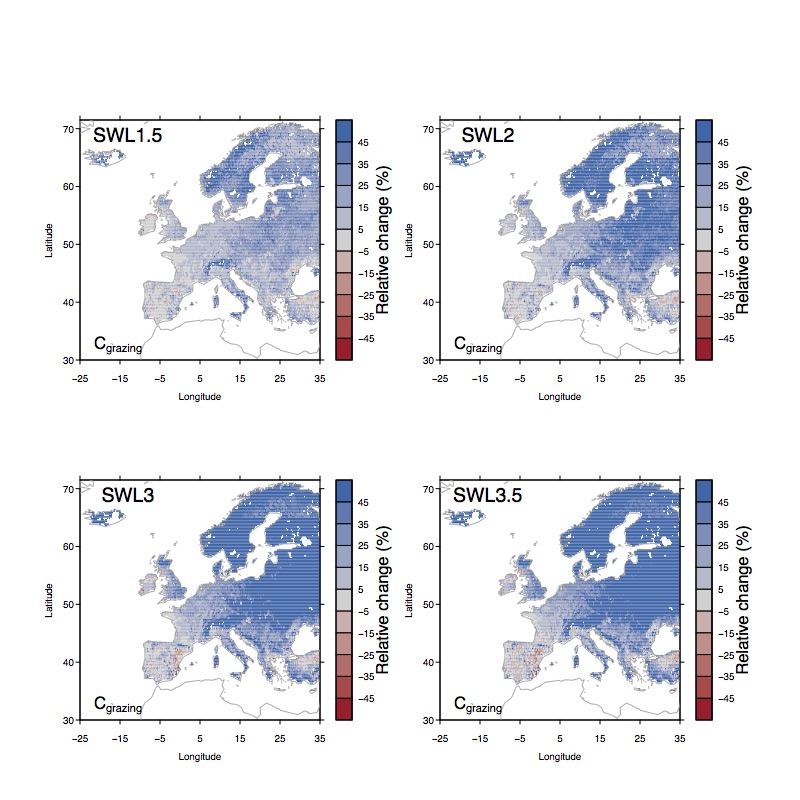


Figure S4. Simulated relative changes of annual grazing capacity (*C_grazing_*) from experiment *E_control_*. The change of *C_grazing_* is defined as the difference between *C_grazing_* during 30 years in the future corresponding to each SWL minus *C_grazing_* in reference historical period (1981-2010).


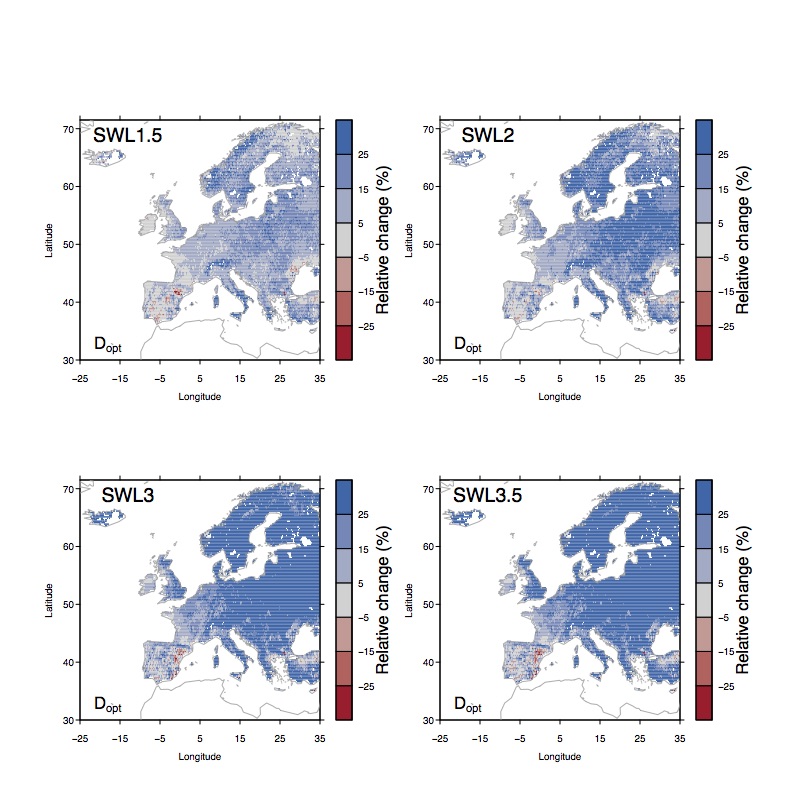


Figure S5. Simulated relative changes of optimal livestock density (*D_opt_*) from experiment *E_control_*. The change of *D_opt_* is defined as the difference between *D_opt_* during 30 years in the future corresponding to each SWL minus *D_opt_* in reference historical period (1981-2010).


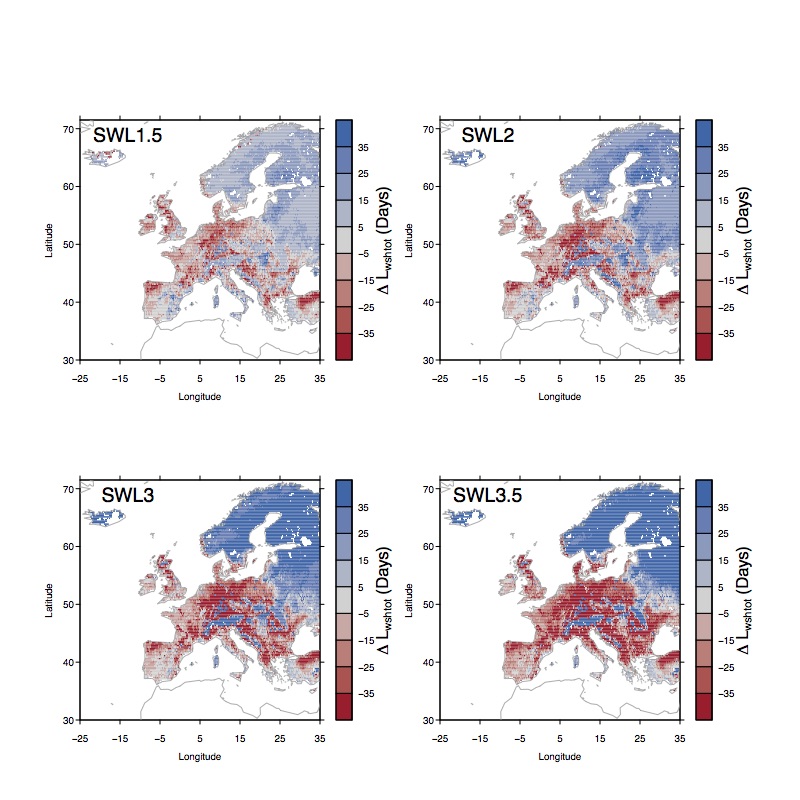


Figure S6. The contributions of the changes in the above-ground biomass availability to the changes in grazing season length (i.e., ∆*L_biomass_*).


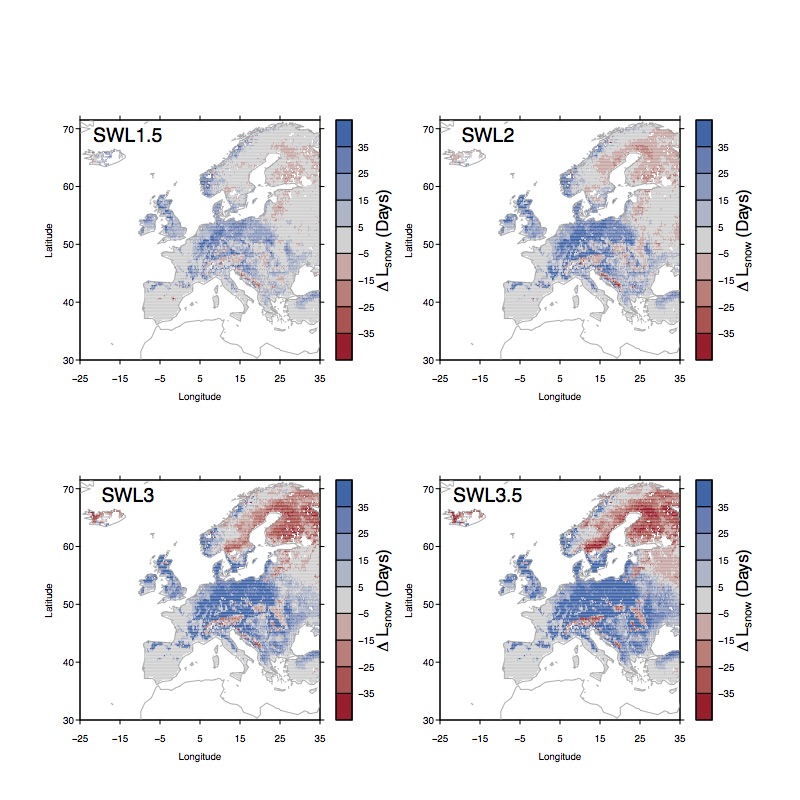


Figure S7. The contributions of the changes in the snow cover days to the changes in grazing season length (i.e., ∆*L_snow_*).


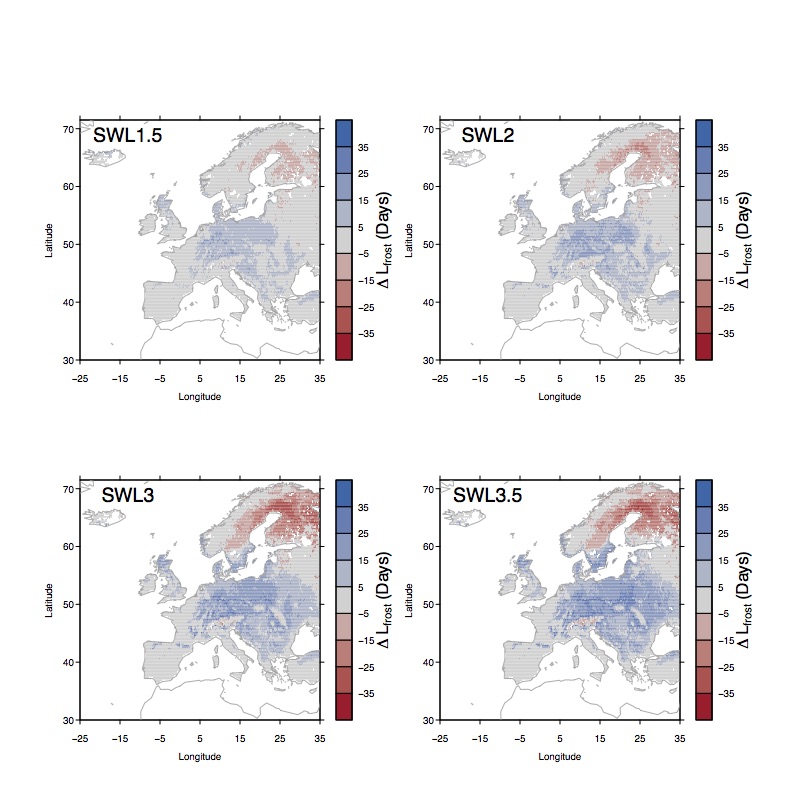


Figure S8. The contributions of the changes in the frost days (air temperature is below 0ºC) to the changes in grazing season length (i.e., ∆*L_frost_*).


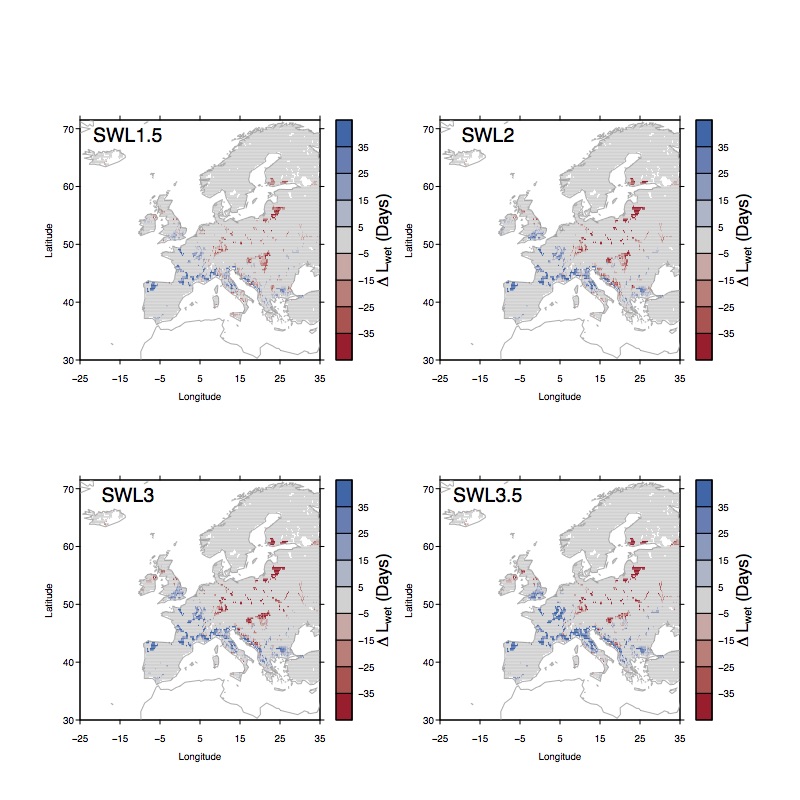


Figure S9. The contributions of the changes in the wet soil days to the changes in grazing season length (i.e., ∆*L_wet_*).


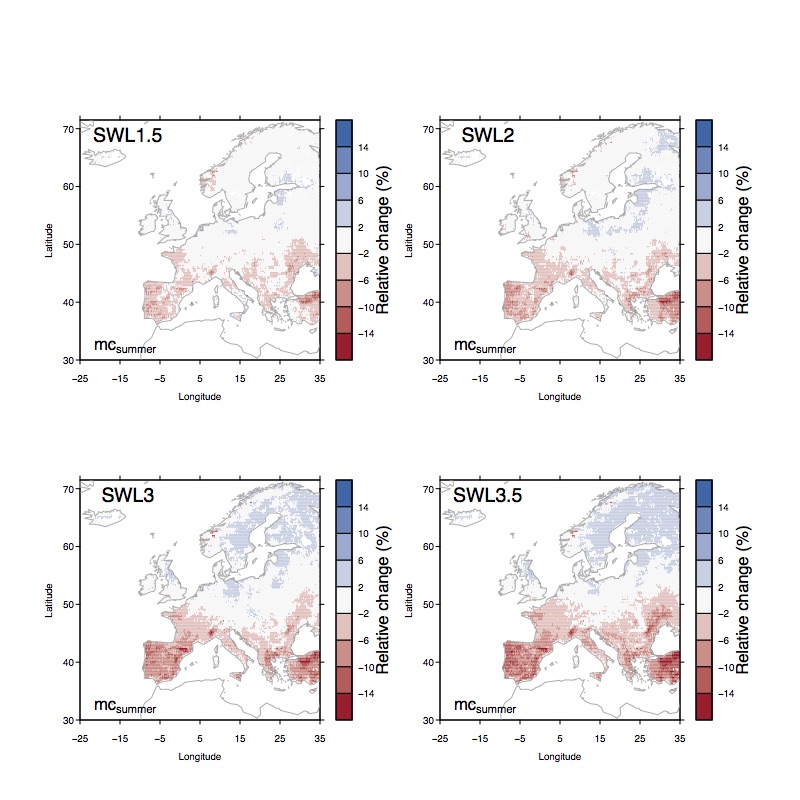


Figure S10. Simulated relative changes of summer mean soil moisture (*mc_summer_*; average over 0-9 cm in depth) from experiment *E_control_*. The change of summer mean soil moisture is defined as the difference between summer mean soil moisture during 30 years in the future corresponding to each SWL minus summer mean soil moisture in reference historical period (1981-2010).


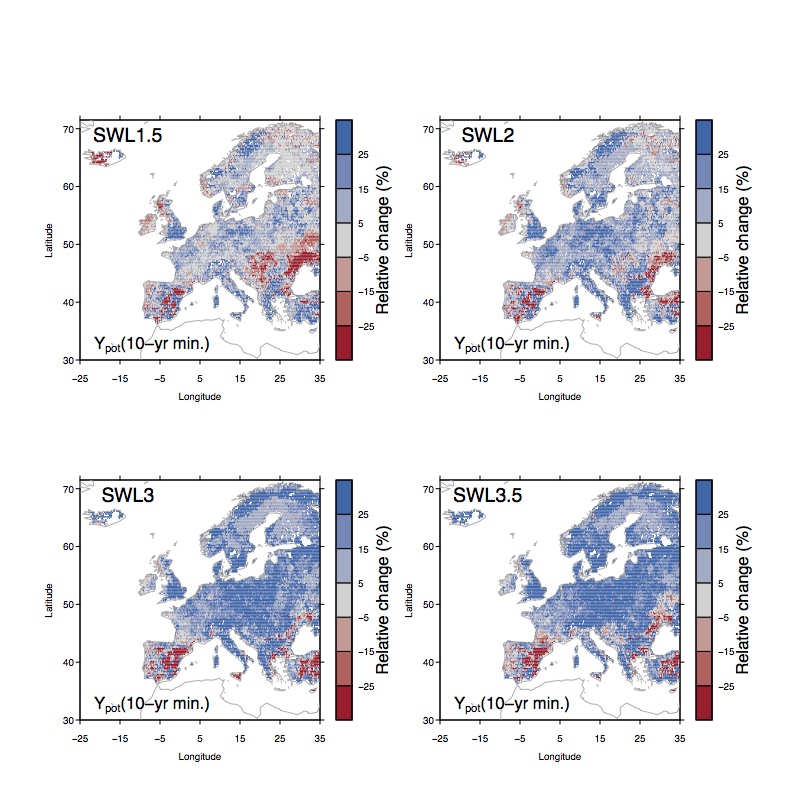


Figure S11. Simulated relative changes of 10-year minimum biological potential productivity (*Y_pot_*) from experiment *E_control_*. The 10-year minimum *Y_pot_* is defined as minimum *Y_pot_* in the past 10 years. The change of 10-year minimum *Y_pot_* is defined as the the difference between 10-year minimum *Y_pot_* during 30 years in the future corresponding to each SWL minus 10-year minimum *Y_pot_* in reference historical period (1981-2010).


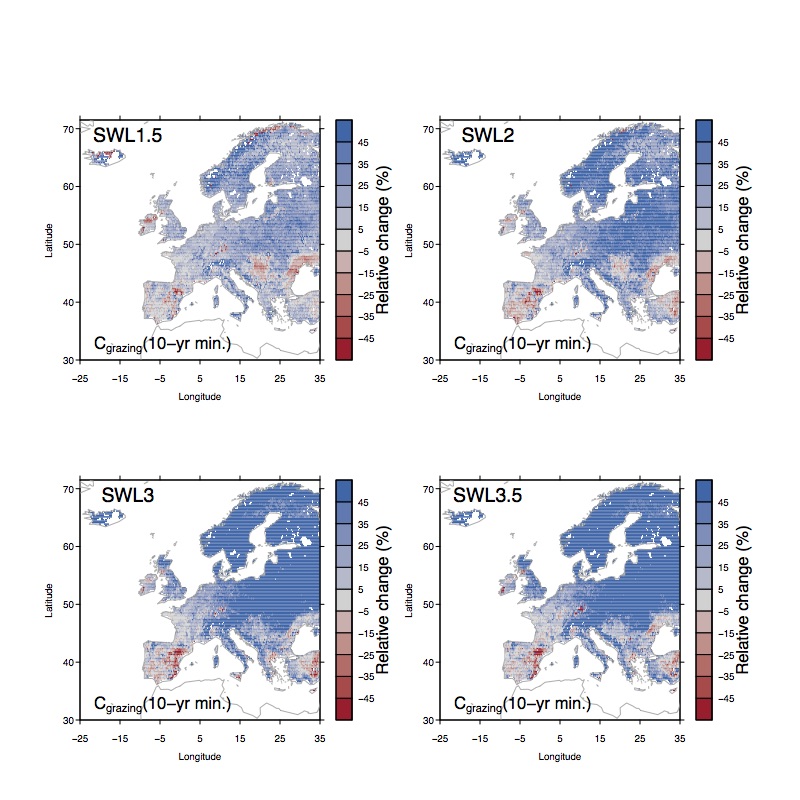


Figure S12. Simulated relative changes of 10-year minimum annual grazing capacity (*C_grazing_*) from experiment *E_control_*. The 10-year minimum *C_grazing_* is defined as minimum *C_grazing_* in the past 10 years. The change of 10-year minimum *C_grazing_* is defined as the the difference between 10-year minimum *C_grazing_* during 30 years in the future corresponding to each SWL minus 10-year minimum *C_grazing_* in reference historical period (1981-2010).


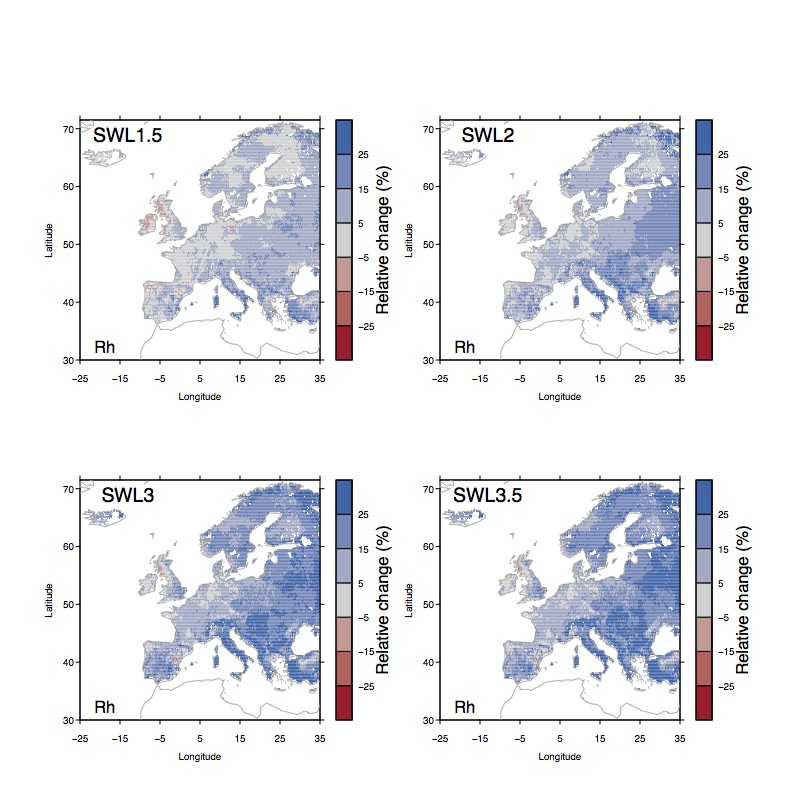


Figure S13. Simulated relative changes of heterotrophic respiration (*Rh*) from experiment *E_control_*. The change of *Rh* is defined as the difference between *Rh* during 30 years in the future corresponding to each SWL minus *Rh* in reference historical period (1981-2010).
